# Nuclear Receptor DHR4 Controls the Timing of Steroid Hormone Pulses During *Drosophila* Development

**DOI:** 10.1371/journal.pbio.1001160

**Published:** 2011-09-27

**Authors:** Qiuxiang Ou, Adam Magico, Kirst King-Jones

**Affiliations:** Department of Biological Sciences, University of Alberta, Edmonton, Alberta, Canada; Stanford University, United States of America

## Abstract

In insects, precisely timed periodic pulses of the molting hormone ecdysone control major developmental transitions such as molts and metamorphosis. The synthesis and release of ecdysone, a steroid hormone, is itself controlled by PTTH (prothoracicotopic hormone). PTTH transcript levels oscillate with an 8 h rhythm, but its significance regarding the timing of ecdysone pulses is unclear. PTTH acts on its target tissue, the prothoracic gland (PG), by activating the Ras/Raf/ERK pathway through its receptor Torso, however direct targets of this pathway have yet to be identified. Here, we demonstrate that *Drosophila Hormone Receptor 4* (*DHR4*), a nuclear receptor, is a key target of the PTTH pathway and establishes temporal boundaries by terminating ecdysone pulses. Specifically, we show that DHR4 oscillates between the nucleus and cytoplasm of PG cells, and that the protein is absent from PG nuclei at developmental times when low titer ecdysone pulses occur. This oscillatory behavior is blocked when *PTTH* or *torso* function is abolished, resulting in nuclear accumulation of DHR4, while hyperactivating the PTTH pathway results in cytoplasmic retention of the protein. Increasing DHR4 levels in the PG can delay or arrest development. In contrast, reducing *DHR4* function in the PG triggers accelerated development, which is caused by precocious ecdysone signaling due to a failure to repress ecdysone pulses. Finally, we show that DHR4 negatively regulates the expression of a hitherto uncharacterized cytochrome P450 gene, *Cyp6t3*. Disruption of *Cyp6t3* function causes low ecdysteroid titers and results in heterochronic phenotypes and molting defects, indicating a novel role in the ecdysone biosynthesis pathway. We propose a model whereby nuclear DHR4 controls the duration of ecdysone pulses by negatively regulating ecdysone biosynthesis through repression of *Cyp6t3*, and that this repressive function is temporarily overturned via the PTTH pathway by removing DHR4 from the nuclear compartment.

## Abstract 2

Pulses of the steroid hormone ecdysone are turned off periodically through nucleo-cytoplasmic oscillations of a nuclear receptor that counteracts the neuropeptide signaling pathway responsible for activating hormone pulses in *Drosophila melanogaster*.

## Author Summary

Steroid hormones play fundamental roles in development and disease. They are often released as pulses, thereby orchestrating multiple physiological and developmental changes throughout the body. Hormone pulses must be regulated in a way so that they have a defined beginning, peak, and end. In *Drosophila*, pulses of the steroid hormone ecdysone govern all major developmental transitions, such as the molts or the transformation of a larva to a pupa. While we have a relatively good understanding of how an ecdysone pulse is initiated, little is known about how hormone production is turned off. In this study, we identify a critical regulator of this process, the nuclear receptor DHR4. When we interfere with the function of DHR4 specifically in the ecdysone-producing gland, we find that larvae develop much faster than normal, and that this is caused by the inability to turn off ecdysone production. We show that DHR4 oscillates between cytoplasm and nucleus of ecdysone-producing cells under the control of a neuropeptide that regulates ecdysone production. When the neuropeptide pathway is inactive, DHR4 enters the nucleus and represses another gene, *Cyp6t3*, for which we show a novel role in the production of ecdysone.

## Introduction

The development of higher organisms is fundamentally dependent on the precise progression of specific gene programs, and even minor differences in the timing of these events can be fatal [1],[2]. The regulation of simultaneous developmental programs generally relies on systemic signals—typically hormones—that coordinate these activities. In most, if not all multicellular organisms, steroid hormones function as precisely timed cues that control diverse gene programs to advance development or synchronize physiological changes. In humans, for example, the onset of puberty—while ultimately under neuroendocrine control—is governed by the action of steroid hormones that coordinate the developmental and behavioral changes associated with sexual maturation [3].

Typically, the release of steroid hormones from their respective glands is temporally controlled, resulting in systemic pulses of defined duration [4]. This raises the interesting question as to how onset, size, and duration of hormone pulses are regulated, since all these variables will affect target tissue responses. The insect molting hormone ecdysone represents an excellent model to address these questions, allowing us to study the dynamic effects of a steroid hormone in the context of a developing organism. In *Drosophila*, major and minor ecdysone pulses (corresponding to high and low hormone titers, respectively) occur throughout development [5]. The major pulses of ecdysone control the molts, the onset of metamorphosis, and the differentiation of adult tissues, while the minor pulses are linked to changes in physiology, such as the termination of feeding behavior. During larval development, ecdysone is mainly synthesized in the PG, and following its release into the hemolymph the hormone is converted to its biologically active form, 20-Hydroxyecdysone (20E), in peripheral tissues [6].

In *Drosophila melanogaster*, at least three minor ecdysone pulses occur in third instar (L3) larvae. These pulses are required for important changes in physiology and behavior, including the commitment of a larva to a pupal fate (critical weight checkpoint), the induction of the *Sgs* (“glue”) genes that serve to attach the pupa to a solid substrate, and the switch from feeding to wandering behavior [7]–[11]. Critical weight is a physiological checkpoint that determines whether the larva has acquired sufficient resources to survive metamorphosis and it commits the animal to undergo puparium formation. Once critical weight is attained, the larva will pupariate within normal time, regardless of whether nutrients are scarce or abundant [12]–[14].

The accurate timing of the major and minor ecdysone pulses suggests that the molecular mechanisms by which the hormone is synthesized, released, and degraded are tightly regulated. The past decade has provided considerable insight into the biosynthetic pathway that converts dietary cholesterol to 20E. Six genes linked to the Halloween mutations encode two different enzyme classes that act in the ecdysone/20E biosynthetic pathway, the cytochrome P450 monooxygenases (*disembodied*, *phantom*, *shadow*, *shade*, and *spook*) [15]–[20], and a short-chain dehydrogenase/reductase (*shroud*) [21]. Two non-Halloween genes have been shown to also play a role in ecdysone synthesis, *neverland*, which encodes a Rieske electron oxygenase [22],[23] and *spookier*, a paralog of *spook* [24]. The first and final biochemical steps of ecdysteroid biosynthesis are reasonably well understood, however relatively little is known about the enzymes that catalyze the intermediate steps, namely the conversion from 7-dehydrocholesterol to 5β-ketodiol. This succession of uncharacterized reactions is generally referred to as the “Black Box” and is believed to harbor the rate-limiting step for the production of ecdysone [25]. Recent work suggests that *shroud* and *spookier* (*spook* in *Manduca* and *Bombyx*) act in the “Black Box” [21],[24], however it is still unknown which enzymatic steps are catalyzed by these enzymes, and whether other enzymes also act in the Black Box.

Some Halloween genes are transcriptionally upregulated when ecdysone levels are high; in *Bombyx* and *Manduca*, for example, the expression levels of *phantom* (*phm*) mRNA correlate well with the two ecdysone peaks preceding pupation [17],[20],[26]. In *Drosophila*, *phm, shadow* (*sad*), and *shade* (*shd*) are induced roughly 12 h before puparium formation, concurrent with the major pulse of ecdysone that triggers this event. However, transcript and protein levels of these three Halloween genes appear to be relatively constant during the first 36 h of the L3 [27], suggesting that the three low-titer 20E pulses are not simply a consequence of modulating gene expression of the Halloween genes. Therefore, it is unclear whether the generation of minor ecdysone pulses involves transcriptional control of any of the known or hitherto unidentified Halloween genes or whether regulation at the transcriptional level plays a role at all. In this study, we show that DHR4 appears to act as a transcriptional repressor of *Cyp6t3*, a cytochrome P450 gene with a previously unknown role in ecdysteroid biosynthetic pathway.

The neuropeptide PTTH is believed to control the timing of all major ecdysone peaks during larval and pupal development [28], however it is unclear whether low-titer ecdysone pulses are also controlled in this manner. Seminal studies conducted in *Bombyx* resulted in the identification of PTTH as a brain-derived neuropeptide, which triggers the production of ecdysone in the PG [29], however the gene encoding PTTH was only recently identified in *Drosophila* [30]. PTTH is sufficient to upregulate some ecdysteroidogenic genes in cultured *Bombyx* PGs [31],[32], and ablation of PTTH-producing neurons in *Drosophila* results in reduced expression of Halloween genes [30]. Surprisingly, ablating PTTH-producing neurons did not abrogate molting or metamorphosis, but instead caused substantial developmental delays with long larval feeding periods that resulted in large animals. This suggests that PTTH acts as a timer that coordinates key developmental transitions and final body size of the developing *Drosophila* larva, but that it is not essential for molting and metamorphosis per se. Interestingly, the same report demonstrated that PTTH mRNA displays an unusual cyclic pattern during the L3, with transcript levels peaking every 8 h. Whether this unexpected transcriptional profile translates into corresponding changes of PTTH peptide levels is unknown, but it is plausible that these PTTH mRNA oscillations are causally linked to the minor ecdysone pulses that occur during the L3 in *Drosophila*.

A recent report showed that *Drosophila* PTTH binds to the Receptor Tyrosine Kinase encoded by the *torso* gene [33]. Torso expression is highly specific to the PG and disruption of *torso* function via tissue-specific RNA interference (RNAi) phenocopies the PTTH ablation experiments. Importantly, the authors also demonstrate that PTTH stimulates ecdysone production through ERK/MAPK, Ras, and Raf, and that loss-of-function of these pathway components (*ERK*, *Ras85D*, and *dRaf*) via RNAi yield large, delayed animals comparable to those seen in the PTTH ablation or *torso* RNAi experiments. Conversely, when a constitutively active form of *Ras* (*Ras^V12^*) [34] was expressed specifically in the PG, larval development was accelerated, resulting in small, precocious pupae.

We previously described a strong hypomorphic mutation in the *DHR4* gene (*DHR4^1^*) that results in prepupal lethality and defects in developmental timing [35]. *DHR4^1^* mutants spend less time feeding than controls and exhibit precocious wandering behavior as well as premature pupariation, ultimately resulting in smaller body sizes. *DHR4* encodes an orphan nuclear receptor most similar to the vertebrate receptor Germ Cell Nuclear Factor [36],[37]. In our original report [35], we found that DHR4 is highly abundant in the cytoplasm of PG cells in mid and late L3 larvae, with little or no detectable protein in the nucleus. We found no DHR4 expression in the two neighboring endocrine tissues of the ring gland (RG), the *corpus allatum*, or the *corpora cardiaca*. We show here that nuclear import of DHR4 is developmentally regulated, and that the protein exhibits a precise nucleocytoplasmic oscillatory pattern in L3 larvae. We also provide evidence that this oscillatory behavior is controlled by PTTH signaling and that DHR4 counteracts the stimulating activity of this neuropeptide by repressing ecdysone pulses. Furthermore, we demonstrate that the accelerated switch from feeding to wandering behavior is consistent with a precocious rise in ecdysone concentrations due to the loss of this repressive function when *DHR4* is mutated or knocked down. Based on RG-specific microarrays, we show that *Cyp6t3* is a cytochrome P450 gene specifically expressed in the RG, and that the gene is normally repressed by DHR4. Further, we demonstrate that *Cyp6t3* plays a key role in ecdysone biosynthesis and that disruption of its function results in low ecdysone titers as well as developmental timing and molting phenotypes. These phenotypes can be rescued by providing an ecdysteroid precursor, 5β-ketodiol, as well as with feeding 20E or ecdysone. We propose a model by which *DHR4* inhibits ecdysone synthesis through the regulation of cytochrome P450 genes, and whereby PTTH activity triggers the translocation of DHR4 from the nucleus to the cytoplasm to temporarily relieve this inhibition, thus allowing for ecdysone pulses to occur.

## Results

### *DHR4^1^* Mutants Display a Range of Growth Defects

**Figure 1:**
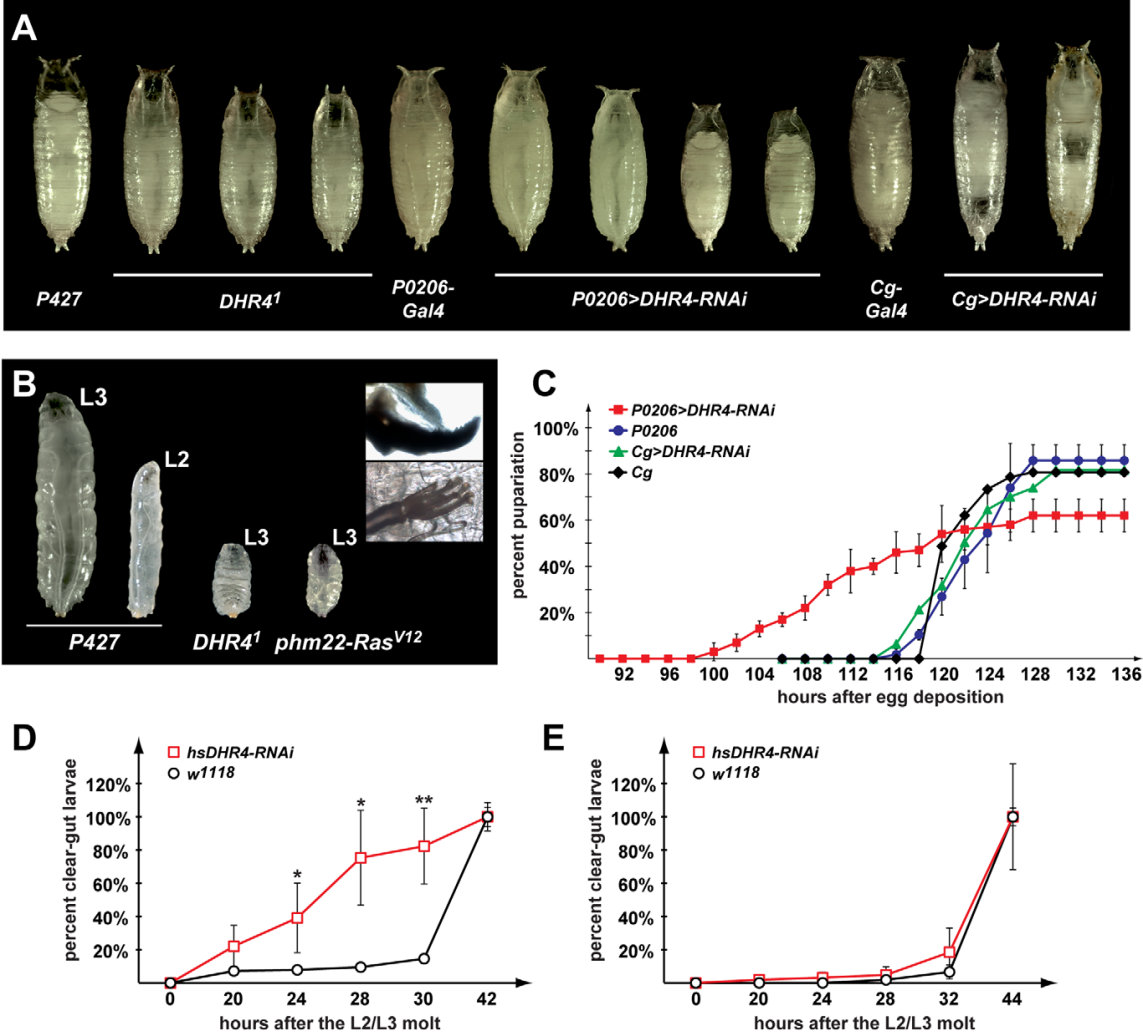
Disruption of *DHR4* ring gland function affects developmental timing. (A) Pupal and prepupal phenotypes include size defects and malformations. From left to right: *P427* parental control line for *DHR4^1^* mutants, *DHR4^1^*, *P0206-Gal4* (x2), *P0206>DHR4-*RNAi (x2) prepupae of various sizes, *Cg-Gal4* (x2) pupa, *Cg>DHR4-*RNAi (x2). (B) Dwarf larvae. *P427* L3 and L2 are controls. A severe growth defect is observed in populations of *DHR4^1^* mutants and *phm22>Ras^V12^* larvae. The two insets show the morphology of mouth hooks and anterior spiracles of a *DHR4^1^* L3 dwarf larva at high magnification. (C) Expression of *DHR4*-RNAi in the RG causes premature pupariation. Percentages of embryos (staged within a 2-h interval) that reached pupariation, hours are after egg deposition. Unpaired Student's *t* tests between *P0206>DHR4-*RNAi (x2) (red, *N* = 386) and *P0206-Gal4* (x2) (blue, *N* = 143) for time points 104 to 118 are all *p*<0.0001 (not indicated in the graph). *Cg-Gal4* (x2) (black, *N* = 150) and *Cg>DHR4-*RNAi (x2) (green, *N* = 251) examine whether timing differences exist when *DHR4*-RNAi is fat body-specific. (D, E) Time course shows percentage of clear gut larvae as a means to measure wandering behavior. Red: *DHR4-*RNAi (*N* = 122, 133 in D and E). Black: *w^1118^* controls (*N* = 157, 115 in D and E). Larvae were heat shocked in late L2 (D) or early L3 (E). *p* values (* *p*<0.05, ** *p*<0.01) are based on Student's *t* test and compare *hsDHR4* and *w^1118^* at the same time point. (C–E) Error bars reflect standard deviation from three to six replicates.

In a previous report, we demonstrated that *DHR4^1^* larvae engage in wandering behavior much earlier than controls, which in turn results in precocious pupariation [35]. We also showed that *DHR4^1^* mutants die as prepupae that are smaller than controls (Figure 1A). Here, we describe an additional growth phenotype—the dwarf larva—that affects ∼5% of the mutant population (Figure 1B). These remarkably small L3 larvae do not feed, are unable to pupariate, and eventually perish as almost transparent larvae due to depleted fat stores (Figure 1B). This extreme growth defect is likely caused by a very early onset of wandering behavior, which is in line with the observation that we consistently observe a small percentage of wandering second instar (L2) larvae in populations of *DHR4^1^* mutants (unpublished data).

In our previous report we also showed that *DHR4^1^* mutants commit earlier to puparium formation than controls. Specifically, when young L3 larvae are transferred to cycloheximide-containing media (which inhibits growth by blocking protein synthesis), *DHR4^1^* mutants are able to pupariate under these conditions, while controls fail to do so, suggesting that the critical weight checkpoint occurs earlier in *DHR4^1^* mutants than controls. This in turn results in early wandering behavior and the ability to pupariate since feeding/cycloheximide uptake has stopped.

These two observations raise the possibility that at least some ecdysone pulses occur precociously in *DHR4^1^* mutants, thus triggering early critical weight assessment, early onset of wandering behavior, and precocious pupariation. *DHR4* is expressed in the PG throughout larval stages and in the fat body prior to molts (Figure S1), raising the question of which of the two tissues is critical for DHR4-dependent regulation of development timing.

### Developmental Timing Phenotypes Are Linked to *DHR4* Expression in the PG

To examine whether it is the expression of DHR4 in the fat body or the PG that is linked to the defects in the timing of wandering behavior and puparium formation, we used the Gal4 system to induce tissue-specific RNAi against *DHR4* in either of these tissues. We found that single copies of the *Gal4* driver and responder transgenes were insufficient to elicit reliable phenotypes. However, when we used lines homozygous for both the driver (RG: *P0206-Gal4*, fat body: *Cg-Gal4*) and the responder (*UAS-DHR4*-RNAi), we found that *DHR4* RNAi in the ring gland triggers phenotypes consistent with early wandering, while fat body-specific RNAi resulted in prepupal lethality in ∼10% of the population (compared to 0% in controls, unpublished data). In particular, *P0206-Gal4* driven *DHR4*-RNAi in the ring gland resulted in early pupariation (Figure 1C) and small precocious prepupae (Figure 1A), while fat body-specific interference of *DHR4* expression yielded phenotypes consistent with a defect in the ecdysone hierarchy, namely prepupal lethality, the failure to evert anterior spiracles (unpublished data), incomplete or absent head eversion, and incorrect location of the gas bubble (Figure 1A). Importantly, we did not observe any timing defects in *Cg>DHR4*-RNAi animals, since we found normally sized pupae that pupariate with similar timing as controls (Figure 1C). This suggests that *DHR4* expression in the fat body is important for prepupal development, while it functions in the PG to control the timing of wandering behavior and puparium formation. The phenotypes observed with fat body and ring gland-specific *DHR4* RNAi lines recapitulate the phenotypes we observed for the *DHR4^1^* mutation, suggesting that *DHR4* function is most critical in these two tissues.

### Precocious Wandering Behavior Is Linked to *DHR4* Expression in Early L3 Larvae

Since critical weight is determined in early L3 larvae, we tested whether *DHR4* function is required during this time to ensure appropriate timing of wandering behavior. Specifically, we used a heat-inducible RNAi line (*hsDHR4*-RNAi) to activate *DHR4* RNAi either 4 h prior to or 4 h after the L2/L3 molt. To examine if either of these heat treatments affected the timing of wandering behavior, we determined the percentage of clear-gut larvae at different time points during the L3 stage. Gut clearing occurs in wandering L3 larvae, typically 30–36 h after the molt and is completed around 4–6 h prior to puparium formation. Although we induced *DHR4* RNAi just 8 h apart, we only observe precocious wandering behavior when the heat treatment is applied in the late L2, but not in the early L3 (Figure 1D,E). In addition, we found a small percentage of dwarf larvae when RNAi was induced in late L2 larvae, while no dwarf larvae were found with the later heat shock (unpublished data). These data suggest that *DHR4* function around the L2/L3 molt is necessary for the correct timing of wandering behavior, which corresponds to the time window when critical weight is determined [38].

### *DHR4* RNAi Triggers Premature Ecdysone Signaling in L3 Larvae

**Figure 2:**
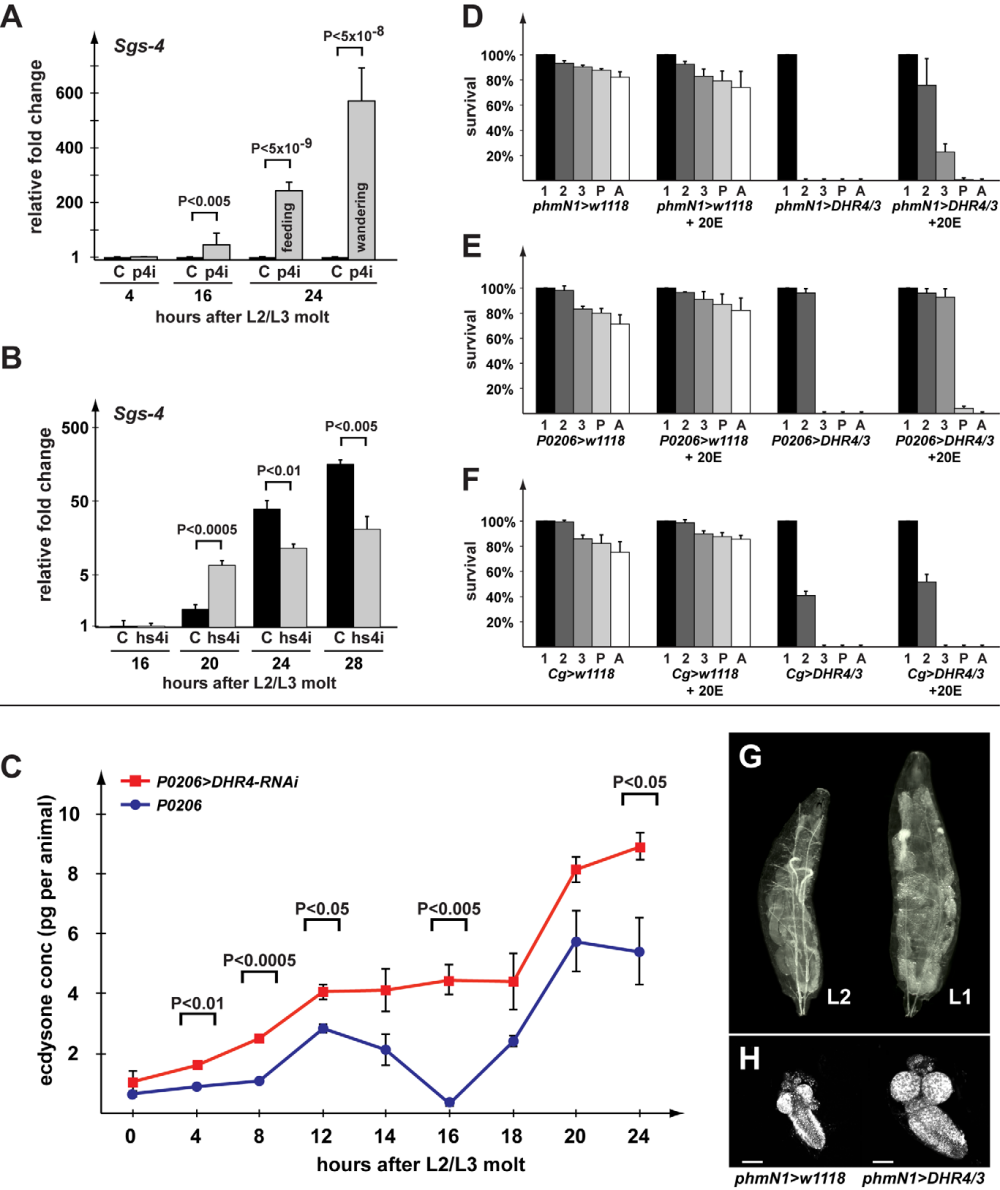
*DHR4*-RNAi affects the timing of ecdysone-mediated responses. (A, B) qPCR of *Sgs4* transcript levels in *P0206>DHR4-*RNAi (p4i) (A) and *hsDHR4-*RNAi (hs4i) (B) animals, hours are relative to the L2/L3 molt, and fold changes are relative to the control of 4-h (A) or 16-h (B) time points. Controls are shown in black. Error bars represent 95% confidence intervals, and *p* values were calculated with the unpaired Student's *t* test. (C) Ecdysteroid measurements during the first 24 h of the third instar. Larvae homozygous either for *P0206>DHR4-*RNAi (red) or *P0206* (blue) were compared. At least three samples were tested per time point, and each sample was tested in triplicate. Error bars represent standard error and *p* values represent results from an unpaired Student's *t* test. (D–F) Percent of larvae reaching indicated stage. 1, L1; 2, L2; 3, L3; P, pupae; A, adults. A starter population of 100 L1 larvae was used for all conditions, each tested in triplicate. *phmN1*, *P0206*, *Cg*: Gal4 transgenes driving expression in the PG, ring gland and fat body, respectively. 20E: 20-Hydroxyecdysone supplemented in the medium. (D–F) Error bars represent standard deviation. (G) *phmN1*>*DHR4/3* cDNA in the PG gives rise to very large L1 larvae (right) when compared to newly molted L2 *phmN1>w^1118^* control larva (left). (H) Central nervous systems (CNS) were isolated from larvae equivalent to those pictured in “G” and stained with DAPI. The scale bars represent 25 µm. (D–H) *DHR4/3: UAS-DHR4* cDNA inserted into the 3^rd^ chromosome.

To test whether early wandering in *P0206>DHR4*-RNAi larvae (Figure 1C) is caused by precocious ecdysone signaling, we examined the expression levels of the glue gene *Sgs-4* via quantitative real-time PCR (qPCR). *Sgs-4* represents one of the first identified ecdysone-inducible genes in *Drosophila* [39],[40]. Roughly 24 h after the molt to the L3 (∼96 h after egg deposition), *Sgs-4* is induced in salivary glands by a low titer ecdysone pulse [10],[11], after which the gene maintains high expression levels until it is abruptly turned off at pupariation. At 24 h after the molt, we observed feeding and wandering larvae in *P0206>DHR4*-RNAi populations, while controls show no sign of wandering behavior. *Sgs-4* expression was drastically higher in the 16 h and 24 h RNAi populations relative to controls, and ∼2-fold higher when one compares the wandering with the feeding larvae in the RNAi group at 24 h (Figure 2A). This indicates that the wandering cohort has received the 20E pulse that induces *Sgs-4* earlier than the feeding cohort. Even at 16 h after the molt, when all *P0206>DHR4*-RNAi larvae are still feeding, we observe much higher expression levels of *Sgs-4* compared to controls, suggesting that the corresponding 20E pulse has occurred already before this time point, thereby preceding the wild type 20E peak (∼20 h after the molt) by at least 4 h.

We also examined whether heat-induced *DHR4*-RNAi in late L2 would trigger precocious *Sgs-4* induction, since this treatment triggers premature wandering behavior (Figure 1D). In contrast to PG-specific *DHR4*-RNAi, we did not observe induction at 16 h after the L2/L3 molt, however at the 20 h mark we found that RNAi larvae had ∼3-fold higher *Sgs-4* mRNA levels than controls (Figure 2B). However, when we examined *Sgs-4* levels at 24 and 28 h after the molt, we found higher expression of the gene in controls, suggesting that *Sgs-4* is induced precociously, but submaximally in *hsDHR4*-RNAi animals. Finally, when heat shocked in early L3, we do not observe differences in *Sgs-4* expression between *hsDHR4*-RNAi and wild type larvae (Figure S2), consistent with our observation that only a heat treatment in late L2 triggers early wandering behavior.

To complement these findings, we analyzed the expression profiles of two isoforms of the *E74* gene, which are both ecdysone-regulated [41],[42]. This approach has been used previously [43] and is based on the idea that the A and B isoforms of the *E74* gene respond inversely to ecdysone concentrations. In particular, if the ecdysone titer is high, *E74A* is induced and *E74B* is repressed, but when ecdysone titers drop to intermediate concentrations, *E74A* is turned off, while *E74B* mRNA levels rise. Therefore, by measuring both isoforms using qPCR, we can infer whether ecdysone concentrations have fallen or risen. When *hsDHR4-RNAi* animals are heat shocked as late L2, we find that *E74A* levels start to rise at 24 h after the molt and remain roughly 2-fold higher than controls at the 24, 28, 32, and 36 h time points, while *E74B* levels show a corresponding drop at 28–36 h (Figure S3, left panels). These findings suggest a preceding rise in ecdysone concentrations, consistent with the precocious induction of *Sgs-4* discussed above. When animals received a heat shock 4 h after the L2/L3 molt, we observed no significant differences in *E74A* and *E74B* transcript levels between *hsDHR4*-RNAi animals and controls (Figure S3, right panels), agreeing with our finding that a later induction of RNAi fails to trigger precocious wandering behavior (Figure 1E).

### *DHR4* Is Required for Repressing Ecdysone Pulses

To test whether the precocious ecdysone signaling in the mid-L3 larvae could be linked to the inappropriate timing and/or duration of a preceding ecdysone pulse, we measured ecdysone titers during the first 24 h of the L3 using a 20E EIA immunoassay (Cayman Chemical). The antibody appears to have similar affinities for 20E and its immediate precursor, ecdysone (E) (Naoki Yamanaka, personal communication), and therefore titers likely reflect a combination of both ecdysteroids. We find that knocking down *DHR4* in the RG overall results in significantly higher ecdysteroid levels at all time points we examined (Figure 2C). Importantly, while we can resolve two minor ecdysteroid peaks in the control, we observe no recession of the first L3 pulse in *P0206>DHR4*-RNAi larvae (Figure 2C, 16 h time point). Rather, the first and the second L3 pulse appear to be fused in RNAi animals, demonstrating that the first pulse was not properly repressed. It is likely that the combination of higher hormone levels and the inability to repress the first pulse causes the premature effects observed for *Sgs-4* and *E74* transcripts, as well as the acceleration of wandering behavior and pupariation.

### Overexpression of *DHR4* in the PG Blocks Molting by Suppressing Ecdysone Pulses

The derepression of ecdysone titers and the premature ecdysone signaling in *DHR4*-RNAi larvae suggest that the wild type function of this nuclear receptor is to inhibit ecdysone production and/or release. To test this idea, we reasoned that increasing *DHR4* expression specifically in the PG using the *phmN1*- and *P0206-Gal4* drivers should maintain low systemic ecdysone levels and prevent ecdysone pulses from occurring. Indeed, we found that *DHR4* blocks molting when overexpressed in the PG, however the penetrance of this phenotype is dependent on the driver/responder combination being used, as well as the chromosomal location of the transgene, suggesting that this effect is dose-sensitive. For instance, when *UAS-DHR4* (inserted on 2^nd^ or 3^nd^ chromosome) is used in combination with *phmN1-Gal4*, animals cannot progress beyond the L1 stage (Figure 2D). Similarly, using *P0206>DHR4/3* (inserted on the 3^rd^ chromosome) results in all larvae being trapped in the L2 stage (Figure 2E), while *P0206>DHR4/2* (inserted on the 2^nd^ chromosome) allows 5%–10% escapers to reach the pupal stage. To test whether the *DHR4*-mediated block in molting could be rescued by ecdysone, we added 20E to the medium and scored the percentage of animals that progress to later stages. Regardless of the driver used, we observed significant rescue when 20E was added to the diet. In the presence of the hormone, ∼80% of the *phmN1>DHR4/3* animals progress to the L2 stage, with another 20% reaching the L3 (Figure 2D). Similarly, most of the *P0206>DHR4/3* animals molt to the L3 in the presence of 20E, and ∼4% of the population pupariate (Figure 2E). Next, we tested whether the ability of *DHR4* to block molting was specific to the PG. Since the *phmN1-Gal4* driver shows some expression in the fat body, we expressed *DHR4* specifically in the fat body using the *Cg-Gal4* driver. Similar to the results obtained with the PG drivers, we observe a developmental arrest in the L1 and L2 stages. In contrast to overexpressing *DHR4* in the PG, however, the developmental arrest caused by fat body-driven expression of *DHR4* cannot be rescued with 20E (Figure 2F). Taken together, these results demonstrate that expressing *DHR4* in the PG blocks molting and interferes with systemic 20E levels in a *DHR4*-dose-dependent manner, where a stronger *Gal4* driver leads to animals trapped in earlier stages.

We further characterized the ability of *phmN1>DHR4* expression to block larval development. Closer examination revealed that these animals survive and continue to grow for up to 10 d as first instar (L1) larvae. These L1 larvae grow very large, accumulate lipids in their fat bodies, and have larger organs than controls due to continued proliferation (Figure 2G,H). This demonstrates that expression of *DHR4* in the PG specifically blocks molting and does not trigger lethality. Rather, it appears that these animals simply lack the ecdysone pulse to molt to the next stage and that all other aspects of larval life function normally.

### The Subcellular Localization of DHR4 Oscillates between Cytoplasm and Nucleus

**Figure 3:**
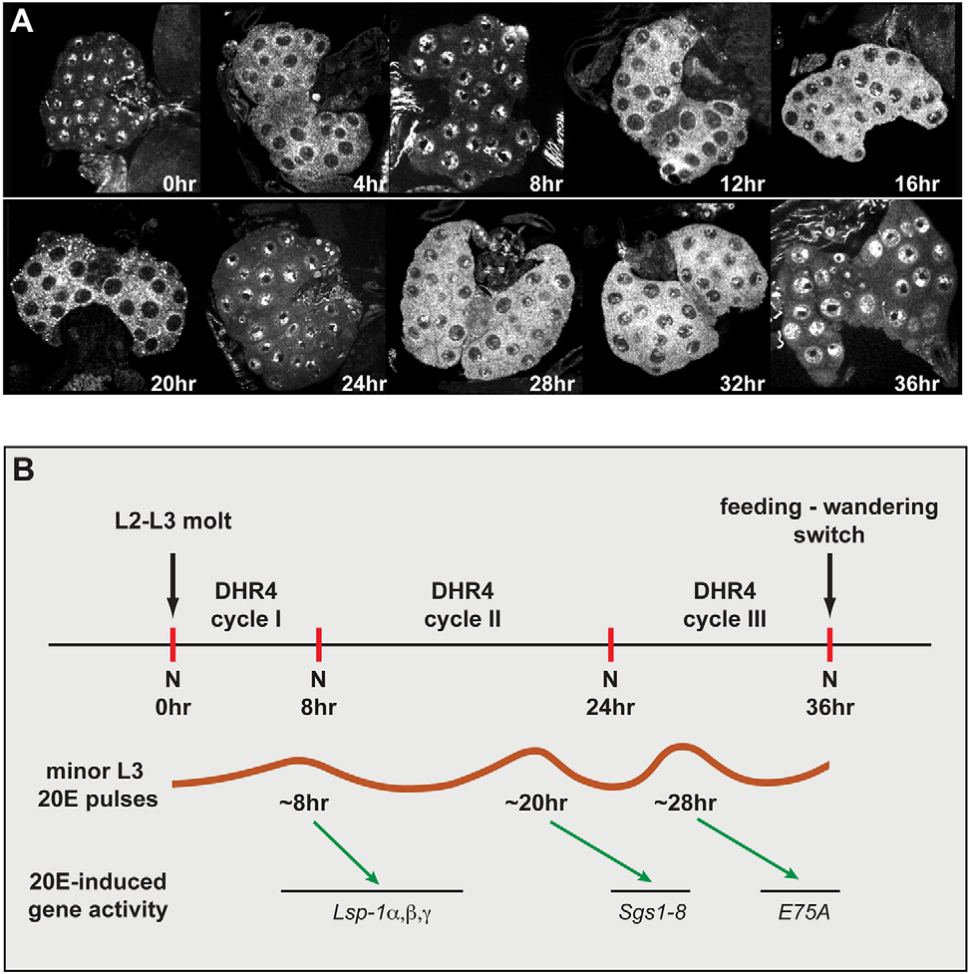
DHR4 oscillates between cytoplasm and nucleus in PG cells of L3 larvae. (A) Confocal images of ring glands isolated from carefully staged *w^1118^* L3 larvae at different times relative to the L2/L3 molt. Ring glands were stained with affinity-purified DHR4 antibody. 15–20 ring glands were tested per time point. (B) Schematic representation of DHR4 oscillations. The three cycles observed in (A) correlate with the appearance of the three minor 20E pulses that are documented for the L3 [8]. These pulses likely induce the *Lsp1*, *Sgs*, and *E75A* genes [11]. N, nucleus.

We previously reported that DHR4 is highly enriched in the cytoplasm of PG cells, even though the protein is nuclear in fat body cells in late L3 larvae [35]. Therefore, it appears that the subcellular localization of DHR4 is differentially controlled in these two tissues, raising the question as to whether DHR4 can enter the PG nucleus at all and, if so, how this translocation is regulated. To test whether DHR4 could enter the nucleus of PG cells at certain times during larval development, we stained ring glands isolated from carefully staged L3 larvae ranging from 0 to 36 h after the molt with affinity-purified DHR4 antibodies. Using this approach, we found that the subcellular localization of DHR4 changes periodically in PG cells during the L3. DHR4 appears to be entirely nuclear at 0, 8, 24, and 36 h, completely cytoplasmic at 4, 12, and 20 h, and present in both compartments at 16, 28, and 32 h after the L2/L3 molt (Figure 3A). During the first 36 h of the L3, DHR4 completes at least three cycles: It shifts from the nucleus to the cytoplasm and back during the first 8 h after the molt, while the next two cycles last 16 and 12 h, respectively (Figure 3B). These three oscillations show an intriguing correlation with the occurrence of the three low titer pulses during the L3. In particular, based on direct measurements of 20E titers [8] and indirect assessments based on ecdysone-regulated gene profiling in L3 larvae [11], the three minor 20E pulses have been mapped to 8, 20, and 28 h after the L2/L3 molt. It should be noted that 20E constitutes the final and active form of the molting hormone, and that a biosynthetic profile in the PG of its immediate precursor, ecdysone, would therefore have to precede the depicted 20E curve. Taking this into account, it appears that DHR4 is cytoplasmic during a minor pulse, but nuclear between these peaks, consistent with the idea that DHR4 regulates the timing of these peaks.

In this context it is also important to note that *PTTH* mRNA was shown to cycle with an 8-h periodicity in staged L3 larvae [30], raising the possibility that a causal link exists between the cyclic behaviors of *PTTH* expression and DHR4 localization. PTTH acts through Ras signaling, and larvae that express constitutively active *Ras* in their prothoracic glands (*phm22*>*Ras^V12^*) display shortened larval stages and small pupae [33], which are strikingly similar to *DHR4* loss-of-function phenotypes. We therefore investigated whether DHR4 acts in the PTTH pathway.

### DHR4 Oscillation Depends on the PTTH Pathway

**Figure 4:**
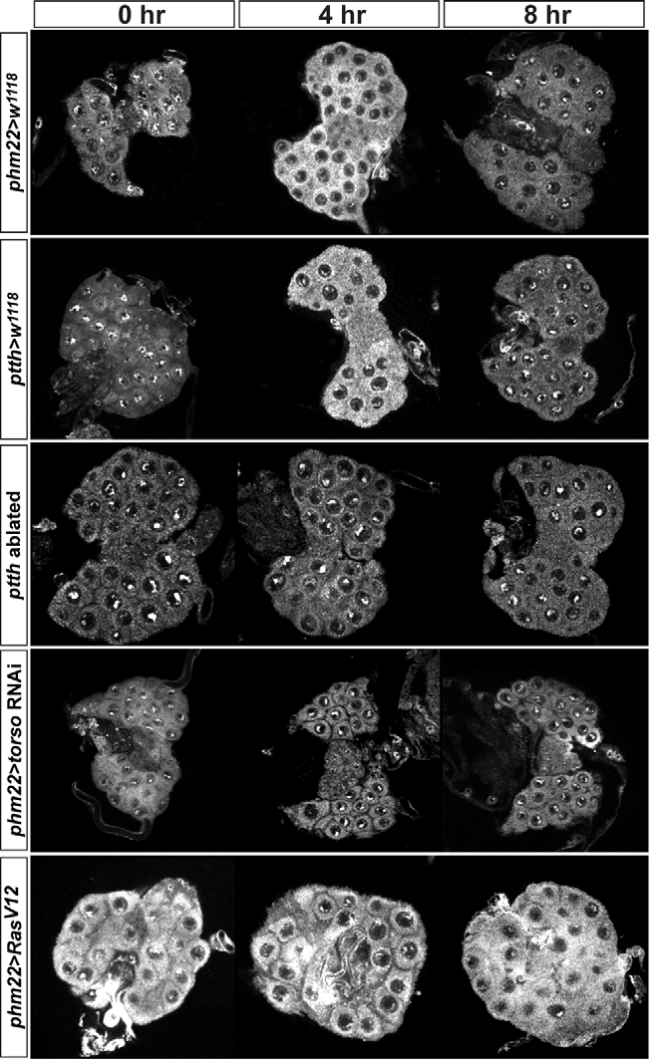
Effects on DHR4 subcellular localization by manipulating PTTH pathway components. DHR4 antibody stains. *phm22>w^1118^* and *ptth>w^1118^* ring glands serve as controls. The *ptth>grim* and *phm22>torso-*RNAi lines disrupt PTTH signaling. *phm22>Ras^V12^* constitutively activates the PTTH pathway. Hours indicate time after the L2/L3 molt. 10–15 ring glands were tested per condition.

To examine the impact of altered PTTH activity on the subcellular distribution of DHR4, we analyzed the location of the DHR4 protein in PGs isolated from 0- to 8-h-old L3 larvae, which were carefully staged at the L2/L3 molt. We reasoned that this stage would not only allow us to follow DHR4 protein through an entire cycle, but also ensure that these animals are as precisely timed as possible. Manipulating components of the PTTH pathway affects developmental timing, which makes later time points difficult to compare between the different genotypes. To test the effects of genetically removing *PTTH* function, we ablated PTTH-producing neurons in *ptth>grim* transgenic animals. In PTTH-abolished animals, DHR4 accumulated in the nucleus, with some residual protein residing in the cytoplasm (Figure 4). Ring glands from later L3 time points look comparable (unpublished data), strongly suggesting that nuclear export and/or degradation of DHR4 is abrogated when PTTH signaling is disrupted. To validate these findings, we examined the effects of *torso* RNAi, which targets the PTTH receptor. Very similar to the PTTH ablation line, we observed nuclear enrichment of DHR4 and loss of oscillation in *phm22>torso-*RNAi animals (Figure 4). We surmised that hyperactivating the PTTH pathway via constitutively active *Ras^V12^* [34] should result in cytoplasmic rather than nuclear accumulation of DHR4. To test this, we carried out DHR4 antibody stains on ring glands isolated from *phm22>Ras^V12^* larvae. In contrast to the nuclear accumulation observed in larvae without intact PTTH signaling, we found strong cytoplasmic enrichment of DHR4 in PG cells when *Ras^V12^* was expressed (Figure 4), indicating that Ras activity dictates whether DHR4 can accumulate in the nucleus or not. Note that constitutively active *Ras^V12^* results in overproliferating PGs, explaining the large and malformed glands we observe.

Taken together, our data demonstrate that the PTTH pathway controls DHR4 and that nuclear accumulation of the protein is only permitted when the pathway is inactive. This suggests that PTTH regulates DHR4 activity by controlling its subcellular localization, thereby permitting or preventing access of DHR4 to its target genes.

### Ras^V12^ Prevents the Nuclear Localization of DHR4 in Fat Body Cells

**Figure 5:**
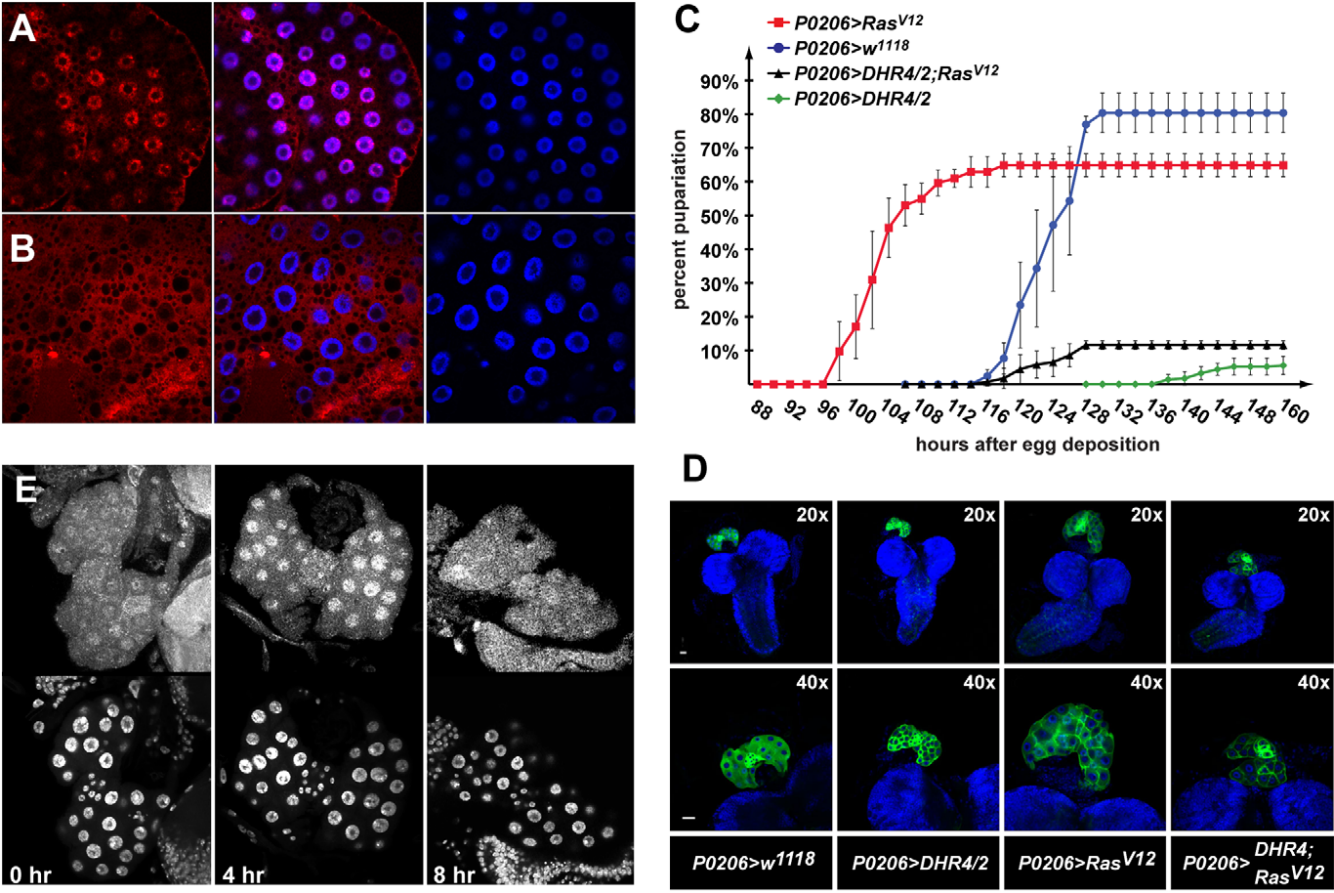
DHR4 acts downstream of Ras and ERK. (A, B) DHR4 antibody stains (red) of *Cg>w^1118^* (A) and *Cg>Ras^V12^* (B) late L2 fat body cells. Blue: DAPI stain of nuclei. (C) Genetic epistasis analysis examining the timing of pupariation for transgenic lines expressing *DHR4* cDNA, *Ras^V12^*, or both. Percentages indicate the fraction of embryos that developed into prepupae at a given time point. All populations were tested in triplicate, total *N* in brackets. Genotypes: *P0206>Ras^V12^* (red, *N* = 151), *P0206>w^1118^* (blue, *N* = 223), *P0206>DHR4/2; Ras^V12^* (black, *N* = 293), and *P0206>DHR4/2* (green, *N* = 265). Error bars represent standard deviation. (D) *DHR4* overexpression inhibits *Ras^V12^*-induced ring gland overgrowth. CNS-RG complexes isolated from early L3 larvae, pictures show same sample at 20× and 40× magnification. Blue: DAPI. Green: *UAS-mCD8-GFP* is recombined to the same chromosome as *P0206-Gal4*, and therefore reflects the expression pattern of the *P0206* driver. Genotypes are listed below the figure. The scale bars represent 25 µm. (C–D) *DHR4/2* denotes *UAS-DHR4* cDNA inserted in the 2^nd^ chromosome. (E) Upper panel: Anti-ERK antibody stains of L3 RGs isolated at 0, 4, and 8 h after L2/L3 molt. Lower panel: Nuclei stain with DAPI. (D–E) 10–15 ring glands were stained per condition.

To further examine the ability of *Ras^V12^* to prevent DHR4 from entering the nucleus, we tested whether Ras^V12^ could abolish DHR4 nuclear localization in larval fat body cells, where DHR4 was shown to be restricted to the nucleus once translation has occurred, at least in L2 and L3 larvae (Figures 5A, S1) [35]. When we specifically drive expression of *UAS-Ras^V12^* in the fat body using *Cg-Gal4*, we found DHR4 to be virtually absent from the nuclei and to be strongly enriched in the cytoplasm instead (Figure 5B), indicating that constitutively active Ras is sufficient to trigger cytoplasmic retention of DHR4. These findings corroborate our results obtained from the PG, providing strong evidence that nucleocytoplasmic distribution of DHR4 is controlled by PTTH signaling.

### *DHR4* Overexpression in the PG Rescues *Ras^V12^* Phenotypes

The dependence of DHR4 on Ras is intriguing because *Ras^V12^* is—to the best of our knowledge—the only other known genetic alteration besides the *DHR4^1^* mutation that results in accelerated larval development and small pupae [33],[43]. We also observe the occurrence of dwarf larvae in *Ras^V12^* animals (Figure 1B). These findings are consistent with our observation that Ras^V12^ prevents DHR4 from accumulating in the nucleus, thus disrupting its nuclear functions, similar to *DHR4^1^* mutants or *DHR4*-RNAi animals. Based on this observation, one would predict that some of the effects of Ras^V12^ could be blocked if one increases the level of DHR4 protein in the same tissue. We therefore asked whether *DHR4* was epistatic to *Ras* or, more specifically, if we could rescue *Ras^V12^*-induced phenotypes by overexpressing *DHR4* specifically in the PG. First, we determined the average time to puparium formation when *Ras^V12^* or *DHR4*, or both together, are expressed in the ring gland using the *P0206-Gal4* driver. As mentioned previously, ∼5%-10% of *P0206>DHR4/2* larvae reach the prepupal stage, allowing us to determine their timing profiles. As previously reported by others, *P0206>Ras^V12^* animals develop much faster than controls [33],[43], preceding the appearance of control prepupae by ∼20 h (Figure 5C). In contrast, *P0206>DHR4/2* prepupae form with a ∼20-h delay compared to controls. However, when *Ras^V12^* and *DHR4* are expressed together in the PG, we observe normal timing of pupariation, and a partial rescue of the DHR4-mediated lethality (Figure 5C).

Since *Ras^V12^* overexpression results in a hyper-proliferation phenotype (Figures 4, 5B), we wondered whether this aspect of Ras activity could also be rescued by *DHR4* overexpression. For this, we examined the morphology of ring glands isolated from staged L3 larvae that express *Ras^V12^* and/or *DHR4*. *P0206>Ras^V12^* larvae have very large ring glands, while *DHR4* expression using the same driver results in slightly smaller ring glands compared to controls (Figure 5D). Importantly, when *DHR4* is co-expressed with *Ras^V12^*, hyper-proliferation of the ring gland appears to be repressed, strongly suggesting that increasing levels of DHR4 in this tissue blocks Ras activity.

### Nuclear Localization of ERK/MAPK and DHR4 Are Inversely Correlated

PTTH acts on the PG by ultimately activating ERK, a MAP kinase, via phosphorylation. Upon activation, ERK can enter the nucleus and phosphorylate nuclear target proteins such as transcription factors or other kinases [44]. Since a key role of PTTH is the induction of ecdysone biosynthesis, and DHR4 appears to have the opposite function, we would predict that PTTH activity would remove nuclear DHR4 via activated ERK entering the nucleus. We therefore stained ring glands from staged early L3 larvae to examine the subcellular localization of ERK at various time points. At 0 and 8 h after the L2/L3 molt, we found ERK evenly distributed between nucleus and cytoplasm, however at the 4-h time point, ERK is strongly enriched in the nucleus (Figure 5E). We conclude that ERK shows an inverse relationship to DHR4, at least at the examined time points: ERK accumulates in the nucleus when DHR4 is enriched in the cytoplasm, and when DHR4 is abundant in the nucleus, we found no particular increase of nuclear ERK over cytoplasmic ERK. These data are consistent with the idea that ERK plays a role in the displacement of DHR4 from the PG nuclei in response to PTTH, in line with our findings that the subcellular localization of DHR4 is regulated by the PTTH/Ras/Raf/ERK signaling pathway.

### *DHR4*-RNAi Ring Gland Microarrays Reveal Misregulation of Cytochrome P450 genes

**Figure 6:**
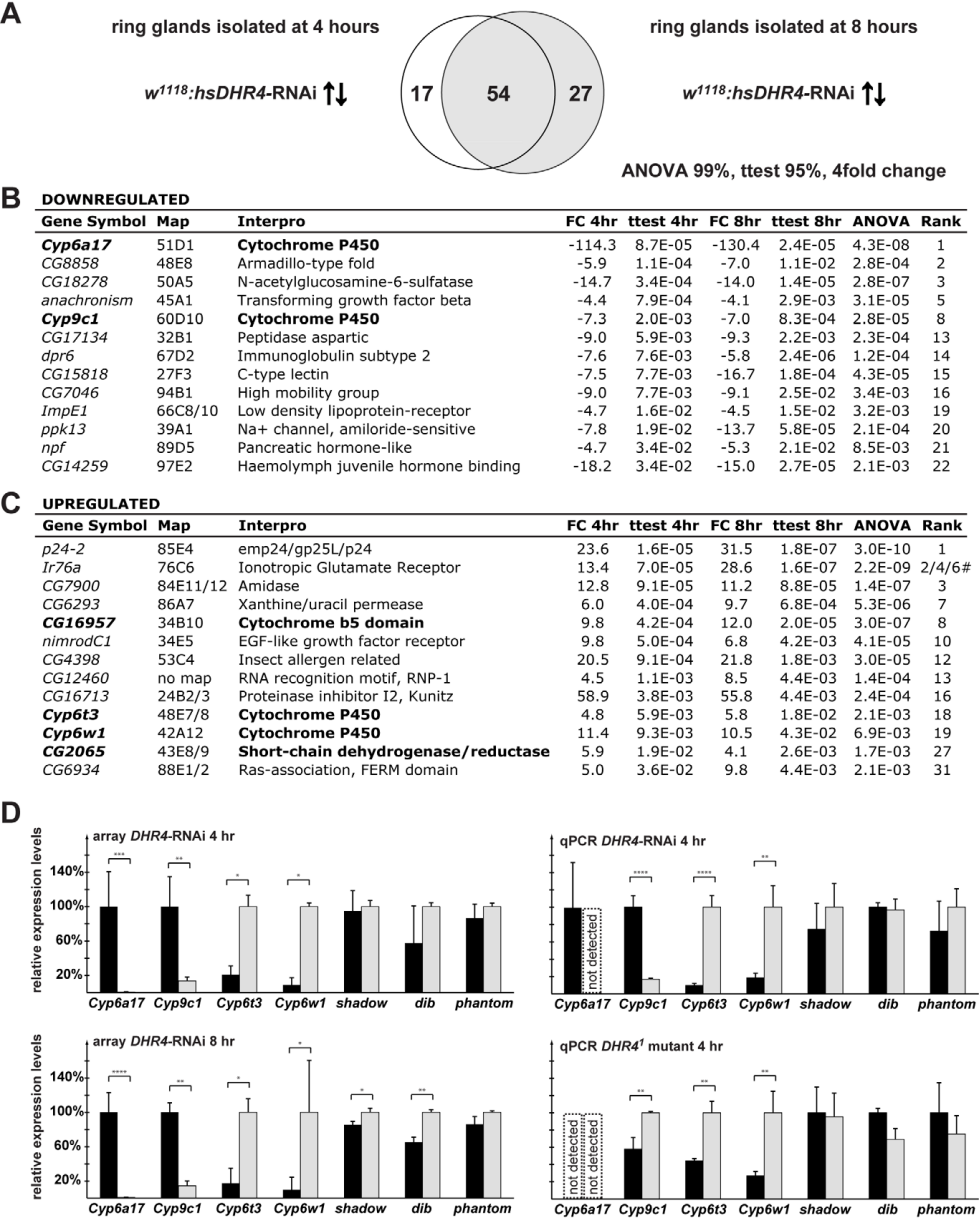
*DHR4-*RNAi ring gland microarray reveals misregulated cytochrome P450 genes. (A) Comparison of microarray data sets representing 71 genes upregulated or downregulated more than 4-fold in 4-h L3 and 81 genes in 8-h L3 *hsDHR4-*RNAi ring glands. Filtering criteria: ≥4-fold change, Student's *t* test with a *p* value of <0.05 for both time points, and an ANOVA *p* value of <0.01. (B, C) Selected genes either downregulated (B) or upregulated (C), sorted by the 4-h *p* value. Genes with possible roles in ecdysone biosynthesis are in bold. # indicates three different probe sets were detected for *Ir76a*. (D) Selected microarray results and qPCR validation in *hsDHR4*-RNAi animals and *DHR4^1^* mutants (grey bars). Controls are shown in black, *w^1118^* for *hsDHR4*-RNAi and *P427* for *DHR4^1^* mutant. *Shadow*, *dib*, and *phantom* failed ANOVA testing at the 95% level, but were included for validation purposes. RNA from brain-ring gland complexes of animals staged at 4-h L3 was used for qPCR validation. Error bars for the array data represent standard deviation, and error bars for qPCR data show 95% confidence intervals. Asterisks indicate significant differences between groups (* *p*<0.05, ** *p*<0.005, *** *p*<0.0005, **** *p*<0.00005 by Student's *t* test).

To identify possible target genes of DHR4, we triggered RNAi in late L2 using *hsDHR4*-RNAi and carried out microarray analysis of ring gland RNA isolated from larvae staged at 4 and 8 h after the molt. To reduce the number of false positives, we analyzed two adjacent time points, 4 and 8 h after the L2/L3 molt, allowing us to select for genes that exhibit significant expression changes at both time points. Using a stringent filtering approach we identified 54 genes whose transcript levels showed a greater than 4-fold difference between controls and *DHR4*-RNAi animals at both time points (Figure 6A). Selected genes from these 54 genes are shown in Figure 6B and C. Intriguingly, among these 54 genes are four cytochrome P450 genes, an enrichment that is highly unlikely to occur by chance (*p* value = 2.4E-11). Two of the P450 genes are downregulated (*Cyp6a17* and *Cyp9c1*), while the other two show increased expression (*Cyp6t3* and *Cyp6w1*), and the effects are very similar between the two time points (Figure 6D, left panels). We also found a short-chain dehydrogenase/reductase (*CG2065*) among the affected genes, which belongs to the same enzyme family as the Halloween gene *shroud*. Finally, *CG16957*, which encodes a protein with a cytochrome b5 domain, is also affected by *DHR4*-RNAi. This protein family is functionally related to cytochrome P450 enzymes because both enzyme classes act as oxidoreductases and carry heme groups.

To validate some of these observations, we analyzed the expression of all four affected cytochrome P450 genes in brain-ring gland complexes isolated from *hsDHR4*-RNAi animals as well as *DHR4^1^* mutants that were staged at 4 h after the L2/L3 molt. We also included the analysis of the Halloween genes *sad*, *dib*, and *phm* as additional controls, in case these genes were affected, but not identified by the microarray approach. As expected, all four cytochrome P450 genes identified by the array display very similar profiles in the qPCR validation experiments (Figure 6D, right panels). When we analyzed the samples derived from the *DHR4^1^* mutants, we confirmed that *Cyp6t3* and *Cyp6w1* are significantly higher when *DHR4* function is impaired. However, we found that *Cyp9c1* displayed higher rather than lower levels compared to controls. In addition, we were unable to detect *Cyp6a17* in *DHR4^1^* mutants or in the corresponding parental line, *P427*, suggesting that both *Cyp9c1* and *Cyp6a17* expression varies substantially between different genetic backgrounds. Future experiments will address whether *Cyp9c1* or *Cyp6a17* are dependent on *DHR4* function. Our qPCR analysis revealed no substantial effects on the tested Halloween genes (Figure 6D, all panels), confirming the microarray results. Taken together, our microarray data identified two cytochrome P450 genes, *Cyp6t3* and *Cyp6w1*, which display significantly higher expression levels in the ring glands of *hsDHR4*-RNAi animals as well as in brain-ring gland complexes isolated from *DHR4^1^* mutants, indicating that DHR4 normally represses these genes.

### *Cyp6t3* Has a Novel Role in the Biosynthesis of Ecdysone

**Figure 7:**
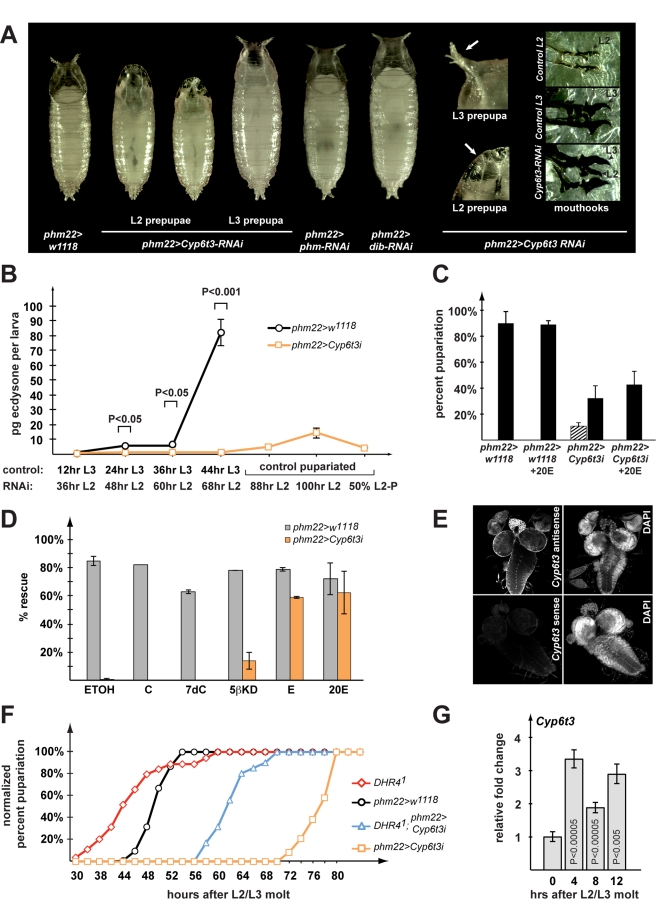
Functional characterization of *Cyp6t3*. (A) PG-specific *Cyp6t3*-RNAi phenotypes VDRC #109703), compared to *phm22>w^1118^* control (left). Insets show the morphology of anterior spiracles and double mouth hooks of *phm22*>*Cyp6t3*-RNAi compared to controls. (B) Whole-body ecdysteroid titer measurements comparing equivalent L2 and L3 stages between *phm22>Cyp6t3*-RNAi animals (orange) and *phm22>w^1118^* controls (black). Time points indicate hours after the L2/L3 molt (control) or after the L1/L2 molt (*phm22>Cyp6t3*-RNAi). For every genotype/time point, 3–4 samples (*N* = 30–45 larvae) were each tested in triplicate. Error bars indicate standard error. (C) Feeding ecdysone to *Cyp6t3-*RNAi larvae rescues L2 pupae phenotype. Percentages of L2 pupae (striped) and L3 pupae (black) of *phm22>w^1118^* and *phm22>Cyp6t3*-RNAi in populations fed a standard medium with or without 20E. Error bars indicate standard deviation, *N* = 150–200 for each condition. (D) Feeding 5β-ketodiol to *Cyp6t3-*RNAi larvae rescues larvae beyond the L2 stage. C424 instant fly medium (Carolina) was supplemented with different ecdysteroid precursors or the carrier alone (ethanol). Percentages show fraction of embryos reaching the L3 stage. Grey: *phm22>w^1118^*. Orange: *phm22>Cyp6t3*-RNAi. Error bars indicate standard deviation, *N* = 150–200 for each condition. ETOH, ethanol; C, cholesterol; 7dC, 7-dehydrocholesterol; 5βKD, 5β-ketodiol; E, ecdysone; 20E, 20-hydoxyecdysone. (E) In situ hybridization of *Cyp6t3* antisense and sense probes. Early L3 larval CNS-RG complexes with eye-antenna imaginal discs were examined at 20× magnification. A DAPI stain of the nuclei is included. (F) Genetic epistasis analysis examining the timing of pupariation in animals carrying a *DHR4^1^* mutation, *phm22-Gal4*//*Cyp6t3*-RNAi transgenes, or both. Percentages were normalized to the final number of pupae for each genotype and represent the fraction of larvae that formed pupae at a given time point. *DHR4^1^* mutants (red, *N* = 54), *phm22>w^1118^* (black, *N* = 180), *phm22>Cyp6t3*-RNAi (orange, *N* = 600), and *DHR4^1^; phm22*>*Cyp6t3*-RNAi (blue, *N* = 107). (G) Transcriptional profile of *Cyp6t3* in early L3. Brain-ring gland complexes were dissected from carefully staged *w^1118^* larvae of indicated time points. Fold changes are relative to 0 h after the L2 to L3 molt. Error bars in (G) represent 95% confidence intervals. *p* values (Student's *t* test) are relative to the previous time point. (B,C,D,F) *Cyp6t3i*: short for *Cyp6t3*-RNAi.

Of the two cytochrome P450 genes reproducibly affected by loss-of-*DHR4* function, *Cyp6t3* and *Cyp6w1*, we chose to analyze *Cyp6t3* in more detail for two reasons. First, *Cyp6t3*, but not *Cyp6w1* (unpublished data), is specifically expressed in the ring gland based on qPCR analysis, which shows ∼20-fold enrichment of *Cyp6t3* transcripts in the ring gland compared to whole body (Figure S4). We confirmed this by *in situ* hybridization, which demonstrates that *Cyp6t3* is specifically expressed in the prothoracic glands and the *corpus allatum* (Figure 7E). Bleed-through of the tyramide-amplified signal did not allow us to determine whether *Cyp6t3* is also expressed in the *corpora cardiaca*. Second, when we examined the changes in gene expression at 0, 4, 8, and 12 h after the L2/L3 molt, we found that *Cyp6t3* levels, but not *Cyp6w1* levels, oscillate during this time window, where lower concentrations of *Cyp6t3* correlate with nuclear DHR4, consistent with the idea that DHR4 represses this gene (compare Figures 7G and S5 with Figure 3A). For these reasons we examined whether interfering with *Cyp6t3* function in the PG via RNAi results in any developmental defects. Specifically, we expressed *Cyp6t3* RNAi (VDRC #109703) [45] in the PG by generating *phm22>Cyp6t3*-RNAi animals. Disrupting *Cyp6t3* in this manner generates phenotypes typically observed in mutants that have defects in the ecdysone synthesis pathway [30],[38],[46],[47]. For instance, we observe very large pupae (similar in size to *phantom* (*phm*) and *disembodied* (*dib*) RNAi pupae, Figure 7A), double larval mouthhooks (a common molting defect, Figure 7A inset), and L2 prepupae (Figure 7A). The latter phenotype occurs when larvae forego the molt to an L3 and directly molt from an L2 to a prepupa. This phenotype is relatively rare and has been only associated with mutations in *E75* [46], *dre4* [48], and *itpr* [49], all of which have dramatically lowered ecdysone levels. We also tested a second independently generated *Cyp6t3* RNAi line (VDRC #30896, Figure S6), which is based on a smaller dsRNA construct. In this RNAi line, we also observe very large pupae, consistent with a longer feeding period, but failed to identify any L2 prepupae. Feeding 20E to these animals completely rescues the large body size (Figure S6). Since the VDRC line #109703 was stronger than the #30896 line, we continued our studies with the former line. No phenotypes are observed when *Cyp6t3*-RNAi is expressed in the fat body (unpublished data), indicating that the phenotypes induced by *phm22*>*Cyp6t3*-RNAi are specific to the PG.

Next, we examined whether *Cyp6t3*-RNAi animals have lower ecdysone titers. To test this, we compared ecdysteroid concentrations at multiple time points between L3 control larvae and delayed L2 larvae of the same absolute age. The latter ultimately develop into L2 prepupae. As expected, *Cyp6t3*-RNAi larvae have severely reduced ecdysteroid titers compared to controls, but generate a small pulse before they form L2 prepupae (Figure 7B, 100 h L2 time point). In addition, we also measured ecdysteroid concentrations in earlier stages and found that *Cyp6t3*-RNAi animals have lower hormone levels at all larval stages, but not as embryos (Figure S7). One would predict that feeding ecdysone to *phm22>Cyp6t3*-RNAi animals should rescue at least some of the phenotypes, and indeed we observed that the occurrence of L2 prepupae is completely rescued when 20E is added to standard medium (Figure 7C), corroborating our finding that *phm22>Cyp6t3* RNAi affects ecdysone production. To further characterize at which step in the ecdysone biosynthetic pathway Cyp6t3 might act, we examined which 20E precursors might also result in a rescue. For this, we took advantage of the fact that the *Cyp6t3*-RNAi phenotype was more pronounced on an instant medium (4–24, Carolina Biological Supply Company), hereafter referred to a “C424.” This medium is naturally low in cholesterol and other sterols, and has been used by us for sterol rescue studies before [50]. On this medium, *phm22*>*Cyp6t3*-RNAi animals very rarely progress beyond the L2/L3 molt (<0.5%), either dying as L2 larvae or L2 prepupae. When we supplemented C424 with carrier only (ethanol), cholesterol, or 7-dehydrocholesterol (7dC), we failed to see any rescue, defined by larvae developing to L3 larvae or later stages. In contrast, adding E or 20E to C424 medium resulted in >60% rescue, while supplementation with 5β-ketodiol rescued ∼15% of the *Cyp6t3*-RNAi population past the L2/L3 molt (Figure 7D), with some animals reaching the pupal stage (unpublished data). The lower percentage of rescued animals with 5β-ketodiol likely reflects the fact that this compound has to enter the PG, while E and 20E can act directly on the target tissues. This strongly suggests that Cyp6t3 plays a role in the black box, since mutations affecting enzymes acting downstream cannot be rescued with 5β-ketodiol [21],[24].

### *Cyp6t3* Is Epistatic to *DHR4*

While our data demonstrate that *Cyp6t3* is specifically expressed in the ring gland, it appears to be expressed at a fairly low level. Based on our microarray and qPCR experiments we estimate that in the ring gland, *Cyp6t3* transcript levels are two orders of magnitude lower than those of *phm*, *dib*, and *sad*. A possible explanation for this is that *Cyp6t3* forms part of a “bottleneck” for ecdysone production and that a low abundance of transcripts renders *Cyp6t3* more susceptible to transcriptional regulation than its Halloween counterparts. We therefore wondered whether (a) overexpression of *Cyp6t3* would be sufficient to alleviate this bottleneck and accelerate ecdysone synthesis and development and (b) if loss of *Cyp6t3* function in a *DHR4^1^* mutant background is necessary for accelerated development of these animals. In the first experiment, we generated a *phm22>Cyp6t3* line that expresses a *Cyp6t3* cDNA at high levels in the PG [51]. This resulted in no obvious phenotypes with respect to timing or overall morphology (unpublished data), suggesting that *Cyp6t3* is not sufficient to accelerate developmental timing via an increased rate of ecdysone production. In the second experiment we found that *DHR4^1^; phm22>Cyp6t3*-RNAi larvae display delayed instead of accelerated development relative to *w^1118^* controls (Figure 7F). This indicates that upregulation of *Cyp6t3* in *DHR4^1^* mutants (Figure 6D) is necessary for the accelerated development in these animals, and that lowering the levels of *Cyp6t3* via RNAi effectively abolishes this effect. We conclude that *Cyp6t3* is necessary but not sufficient for accelerating development, suggesting that if *Cyp6t3* is indeed part of a bottleneck, it is not acting alone in this rate-limiting step.

## Discussion

### DHR4 Oscillation and Its Dependence on PTTH Signaling

**Figure 8:**
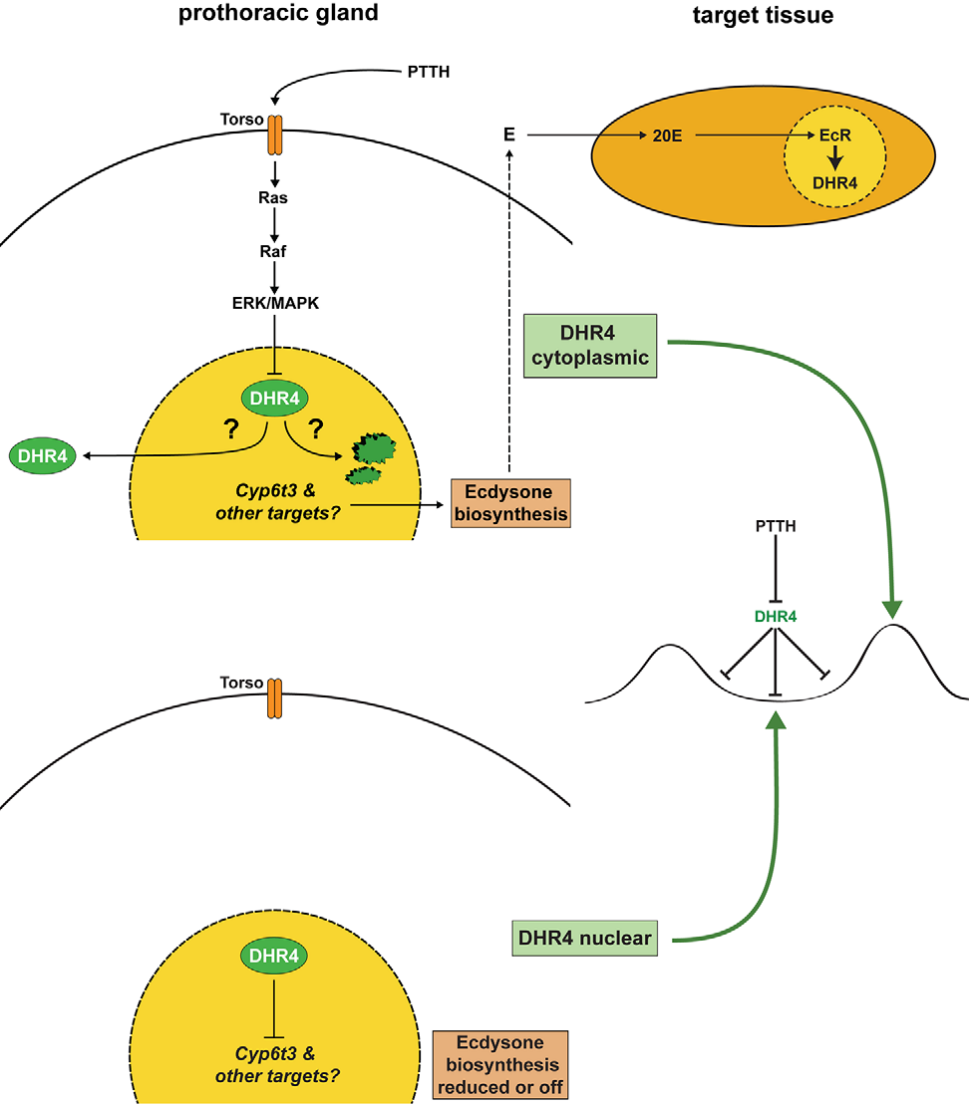
Models for DHR4 function. DHR4 represses ecdysone pulses dependent on whether PTTH signaling is active or inactive (middle panel). In the presence of PTTH signaling (upper panel, left), DHR4 is removed from the nucleus either by shuttling to the cytoplasm or by protein degradation, which allows for ecdysone biosynthesis to occur. In the absence of PTTH (lower panel), DHR4 remains in the nucleus and represses *Cyp6t3* and possibly other genes with roles in ecdysone production, thereby lowering ecdysone titers. During molts, DHR4 acts as a component of the EcR-controlled gene hierarchy in some target tissues of the hormone (upper panel, right). See text for details.

A series of reports have provided ample evidence that PTTH utilizes the Ras/Raf/ERK pathway to regulate ecdysone biosynthesis in *Bombyx*, *Manduca*, and *Drosophila* [33],[52]–[56]. In the present study, we demonstrate that a critical readout of this pathway is the nuclear receptor DHR4. We suggest a simple model where PTTH represses DHR4 activity via its removal from the nucleus, while DHR4 in turn represses the occurrence of low-titer ecdysone peaks when nuclear (Figure 8). In laboratory fly cultures, *PTTH* was shown to be a non-essential gene, however when PTTH-producing neurons are ablated, development is substantially delayed and animals have a concomitant increase in body size [33]. We show here that disrupting *DHR4* specifically in the PG results in opposite phenotypes to loss-of-*PTTH* function, where animals are smaller and develop faster than controls. Like PTTH ablation lines, animals homozygous for *P0206>DHR4*-RNAi can be kept as a viable stock, consistent with the idea that DHR4 functions in the PG as a PTTH-dependent, non-essential developmental clock to generate appropriately timed ecdysone pulses. It is of interest to note that the expression of constitutively active Ras in the PG of *P0206>Ras^V12^* animals results in accelerated larval development and small pupae, very similar to *DHR4^1^* mutants and *P0206>DHR4*-RNAi animals. *P0206>Ras^V12^* animals are also viable, and we showed that L3 larvae of this genotype accumulate DHR4 in the cytoplasm of PG cells (Figure 4). This strongly suggests that *P0206>Ras^V12^* larvae display these phenotypes precisely because DHR4 protein is prevented from entering PG nuclei, thereby mimicking the loss-of-function phenotypes observed in *DHR4* RNAi or mutant larvae.

We have demonstrated that the PTTH pathway controls the subcellular location of DHR4. Loss of PTTH signaling results in nuclear presence of DHR4, while constitutively activating this pathway leads to cytoplasmic localization of the protein. It is unclear at this point whether the DHR4 oscillations represent shuttling or involve cycles of degradation and synthesis. Shuttling would require a stable DHR4 protein that moves in and out of the nucleus, however this would be difficult to reconcile with the fact that *DHR4* RNAi works well in our hands, since a continuously shuttling protein would be impervious to RNAi. It is evident that sufficient turnover of the DHR4 protein must occur, at least around the L2/L3 molt, when we conducted our heat-induced *DHR4*-RNAi experiments (Figures 1D–E, 2B, and S3). This raises the question of whether *DHR4* mRNA levels are oscillating, given that the degraded protein must be replaced periodically. When we conducted a time course microarray of wild type ring glands, we observed very low and constant levels of *DHR4* mRNA, which does not support the idea that *DHR4* transcripts levels are oscillating (Ou et al., manuscript in preparation). Based on these data, we suspect that *DHR4* mRNA is highly stable in PG cells and translated when needed in L3 larvae. Alternatively, our current approach might be too insensitive to detect periodic changes in *DHR4* mRNA levels.

It was shown in mammalian cell cultures that the ERK pathway controls the subcellular localization of the nuclear receptor PPARγ. Specifically, mitogenic stimulation of resting cells causes the binding of nuclear MEK1 to PPARγ, which is followed by rapid export of the protein complex from the nucleus [57],[58]. Our study does not provide direct evidence that DHR4 is phosphorylated. However, our data are suggestive of the idea that ERK plays a role in removing DHR4 from the nucleus, since we found that ERK changes its nucleo-cytoplasmic distribution in early L3 larvae, in an apparent inverse relationship to DHR4, consistent with the notion that it acts upstream of this nuclear receptor (Figure 5E). Future experiments will have to examine whether DHR4 is a direct target of ERK and whether phosphorylation plays a role in the nucleo-cytoplasmic oscillations of DHR4.

An intriguing finding is that *Drosophila PTTH* mRNA levels oscillate with an apparent 8-h cycle time throughout the 48-h duration of the L3 stage [30]. DHR4, on the other hand, displays an 8-h, 16-h, and 12-h cycle time for the first 36 h of the L3 stage, raising the question as to how these ultradian periods are established. What could account for the difference in these cycle times? A simple possibility is that we were unable to detect all DHR4 cycles and that the DHR4 oscillations are well aligned with the PTTH cycles. In this study, we chose to conduct a time course based on a 4-h step size, because we consider 4 h a robust time interval that should compensate for the inherent asynchrony that exists in developing *Drosophila* larvae. We examined 15–20 ring glands per time point, and only found some discrepancies among ring glands from later time points, likely due to the asynchronous development of the population. However, it appears that during some time points, such as 16 h after the L2/L3, DHR4 is detected in the nucleus and the cytoplasm, and it is possible that this reflects a transition phase of a cycle we might have missed. Future studies could attempt a time course with a 2-h step size, ideally in combination with a *Sgs3-GFP* reporter line to re-stage animals in the mid third instar [8].

Alternatively, the differences in cycle duration between DHR4 and PTTH could reflect the possibility of another cyclic process that may contribute to the timing of DHR4 periodicity. An attractive possibility is that circadian rhythms are superimposed on the PTTH oscillations to determine DHR4 cycle times. Anatomical evidence indicates that the central circadian pacemaker cells found in the *Drosophila* brain, the Lateral Neurons, indirectly innervate the PG [59]. A critical effector of these neurons is the neuropeptide PDF (Pigment Dispersing Factor), and a mutation in *pdf* alters the periodicity of PTTH mRNA oscillations [30]. Future experiments will address whether PDF and other components of the circadian clock impinge on the oscillatory behavior of DHR4 in PG cells.

### PTTH and the Transcriptional Control of Ecdysone Biosynthetic Genes

The mechanism by which PTTH regulates ecdysone biosynthesis has been the subject of intense research for the last 35 years. PTTH triggers a complex array of signaling events in the PG that precede the synthesis of ecdysone. These include second messengers like Ca^2+^ influx and the synthesis of cAMP, followed by the stimulation of Protein kinase A, the activation of p70S6K and the concomitant phosphorylation of ribosomal protein S6, a myriad of tyrosine phosphorylations, as well as the activation of the ERK pathway discussed in this report [56]. Clearly, PTTH triggers a range of events, which, among others, results in the transcriptional upregulation of genes required for ecdysteroid biosynthesis. The first evidence that PTTH is sufficient to increase transcript levels of an ecdysteroidogenic gene is based on the observation that the *Bombyx disembodied* gene (*dib-Bm*) is upregulated by administering PTTH to cultured prothoracic glands [32]. The upregulation of *phm* and *spo* appears to be more moderate [31],[32], while *sad* transcription appears not to be induced under these conditions [32]. Attempts to generate functional recombinant *Drosophila* PTTH have so far been unsuccessful [33], but loss-of-function studies have confirmed a role for PTTH in the transcriptional regulation of ecdysteroidogenic genes. Specifically, ablation of PTTH-producing neurons resulted in a strong reduction of *Drosophila dib* (∼10-fold down) and had a moderate effect on *phm*, *sad*, and *spok* (2–3-fold down). While similar studies with a *torso* RNAi line have not been published, indirect results come from a recent paper where the authors knocked down *dSmad2* function in the PG, which results in a strong downregulation of *torso* [60]. Concomitantly, *spok* and *dib* are strongly reduced in *phm>dSmad2*-RNAi larvae, but similar to the above findings, no effect was seen for *phm* and *sad*. Taken together, the findings from *Bombyx* and *Drosophila* seem to suggest that the transcriptional effect of PTTH is most clearly established for *dib*, while *phm* and *sad* appear to be less dependent on PTTH signaling. One aspect of PTTH-mediated transcriptional regulation that has not been satisfactorily addressed is whether some of the ecdysteroidogenic genes require PTTH to reach high expression levels in the first place or whether the hormone provides a “boost” to elevate transcript levels even further. According to our RG microarray and qPCR data presented here, we conclude that expression levels of the Halloween genes are very high in L3 larvae, comparable to that of ribosomal genes. We also conducted a microarray time course (Ou et al., manuscript in preparation), which suggests that the Halloween genes are expressed at very high levels during the first 36 h of the L3, without much fluctuation in their expression levels. Therefore, it would seem unlikely, at least according to our data, that transcriptional downregulation of the highly expressed ecdysteroidogenic genes like *dib* or *phm* can be the mechanism by which the three minor ecdysone peaks are generated, which is in line with our finding that knocking down DHR4 in the RG has no effect on *phm*, *sad*, and *dib* (Figure 6D). Rather, it appears that DHR4 negatively regulates *Cyp6t3* and possibly other uncharacterized genes that play critical roles in the production of ecdysone (Figure 8).

DHR4 appears to mainly act as a repressor [35], similar to what has been reported for its vertebrate ortholog GCNF [61]. It remains to be seen whether the genes that are downregulated in our *hsDHR4*-RNAi microarrays are indirect targets of DHR4 or whether this nuclear receptor can act as an activator on some promoters. We have previously shown that DHR4 acts downstream of the 20E receptor EcR as a key component of the ecdysone hierarchy during puparium formation [35]. It therefore appears that DHR4 acts upstream of ecdysone in the PG, but downstream of the hormone in target tissues (Figure 8), which nicely reflects the duality of *DHR4^1^* mutant phenotypes: the mutation affects developmental timing (due to its role in the PG) and puparium formation (due to its role in the fat body).

While some transcription factors have been identified for their roles in the ecdysone production pathway, none have been reported to repress ecdysteroidogenesis, and none have been directly linked to PTTH. A mutation in the *woc* (*without children*) gene, which encodes a putative zinc finger transcription factor, causes low ecdysone levels. This phenotype can be rescued with feeding 7-dehydrocholesterol (7dC), suggesting that Woc might control the conversion of cholesterol to 7dC by regulating the genes required for this step [62]. The nuclear receptor βFTZ-F1 is expressed in the PG of late L3 larvae and is required for normal levels of Phm and Dib protein [27]. Another nuclear receptor, E75A, acts in a feed-forward loop to maintain normal ecdysone levels, possibly by acting upstream of βFTZ-F1 in the PG [46].

### *Cyp6t3* Is a Downstream Target of DHR4

Our ring gland microarray and qPCR data revealed that *Cyp6t3* transcript levels are significantly elevated in *hsDHR4* RNAi animals and *DHR4^1^* mutants. In addition, we showed that *Cyp6t3* expression levels oscillate during the first 12 h after the L2/L3 molt, with lower levels of *Cyp6t3* when DHR4 is nuclear (Figure 7G). It therefore appears plausible that *Cyp6t3* is a direct transcriptional target of DHR4, however direct evidence for this is lacking. No DNA recognition sites have been identified for DHR4, nor is it known whether this nuclear receptor acts as a homodimer, heterodimer, or monomer. According to our microarray data, as well as judging by the cycle numbers required to detect *Cyp6t3* in qPCR experiments, we estimate that transcript levels of this gene are relatively low, probably by two orders of magnitude lower than the Halloween mRNAs for *phm*, *dib*, *spookier*, and *shadow* in PG cells (unpublished data). This is consistent with the finding that *Cyp6t3* was previously neither detected by *in situ* hybridization in any larval tissue, nor amplifiable from larval cDNA, while the aforementioned 4 Halloween genes showed strong expression under the same conditions in the PG of *Drosophila* larvae [63]. However, using tyramide amplification coupled to in situ hybridization, we were able to validate the ring gland-specific expression of *Cyp6t3* (Figure 7E). According to our wild type microarray study, *Cyp6t3* is one out of nine cytochrome P450 transcripts that have a higher than 10-fold enrichment in the ring gland compared to the whole body signal (Ou et al., manuscript in preparation), supporting the idea that this gene has an important role in ecdysteroid biosynthesis.

The fact that *Cyp6t3* is expressed at very low levels raises the possibility that the Cyp6t3 enzyme is scarce and therefore rate-limiting with respect to the production of ecdysone. An attractive model is that DHR4-mediated repression of *Cyp6t3* suffices to reduce ecdysone production to basal levels. If true, one would predict that Cyp6t3 turnover is controlled so that transcriptional control of the *Cyp6t3* gene becomes a relevant factor in controlling ecdysone synthesis. Conversely, derepression of *Cyp6t3* due to loss-of-*DHR4* function could result in faster accumulation of ecdysone, which would account for the timing defects we observe. However, when we overexpressed a *Cyp6t3* cDNA specifically in the PG, we did not observe any obvious phenotypes or effects on development timing, suggesting that changing levels of *Cyp6t3* alone is not sufficient for this response (unpublished data). In contrast, we could show that *Cyp6t3* function is necessary for the accelerated developmental phenotype of *DHR4^1^* mutants, strongly supporting the notion that *Cyp6t3* is a key target of DHR4-mediated repression of ecdysone pulses (Figure 7F).

The degree by which *Cyp6t3* is repressed might directly correspond to the amount of DHR4 protein allowed to enter the nucleus. In support of this idea we find that overexpression of *DHR4* cDNA in the PG results in varying degrees of larval arrest, depending on the strength of the Gal4 driver being used (Figure 2D–F), which in turn suggests that the strength of the phenotype depends on how much DHR4 can enter the nucleus. Therefore, it is conceivable that the nuclear functions of DHR4 are dose-sensitive, giving rise to the idea that the oscillations of this nuclear receptor do not necessarily represent an all or nothing response, but may in fact fine-tune the expression levels of target genes instead.

An interesting question is whether *Cyp6t3* has clearly identifiable orthologs in other insects species, which is the case for the Halloween genes [64]. To address this question we conducted a series of BLAST searches with the *Drosophila melanogaster* Cyp6t3 amino acid sequence as a query. We limited our search to insect species with sequenced genomes and aligned the top hits from this search (Figure S8) and analyzed their phylogenetic relationship using the program Seaview (Figure S9) [65]. Due to the large size of the Cyp450 family 6 [66] and the sequence similarities within this family, we were not able to identify a definitive *Cyp6t3* ortholog outside the *Drosophila* genus. We reached this conclusion based on a reverse BLAST search strategy, which revealed that the top hits from the *D. melanogaster* query did not retrieve Cyp6t3 as the best hit when used as queries themselves. Despite this, Cyp6t3 is highly similar to sequences found in other insect species, and it is likely that very similar paralogs are masking the true “functional” ortholog. Often, reverse BLAST searches fail to reveal definitive orthologs. For instance, the best characterized nuclear receptor in *Drosophila*, EcR, is most similar to both FXR and LXR, depending on whether one uses the DNA-binding domain or the ligand-binding domain as a query [67]. Future studies in other insect species will have to address the question of whether *Cyp6t3* orthologs are also important for ecdysone production.

## Materials and Methods

### Drosophila Stocks

*w^1118^* (#3605) and *UAS-Ras^V12^* (#4847) were ordered from the Bloomington stock center. Gal4 drivers were obtained from labs indicated by the references. Ring gland: *P0206-Gal4*, *UAS-mCD8-GFP* [68], *phm22-Gal4* [33]; *phmN1-Gal4* [69]. Fat body: *Cg-Gal4* [70]. PTTH-Gal4 driver and PTTH ablation line: *UAS-Grim/CyO-act-GFP & ptth-Gal4/Ser-act-GFP* [30]; *DHR4^1^/FM7h,* & *hsDHR4-RNAi* [35]; RNAi lines were obtained from Vienna *Drosophila* RNAi Center [45]. *UAS-Torso-RNAi* (VDRC #4300 & #1016); *UAS-Cyp6t3*-RNAi (VDRC #109703 & #30896); *UAS-phm*-RNAi: (VDRC #100811), *UAS-dib*-RNAi (VDRC #101117).

### Developmental Timing Analysis

Before embryos were collected on grape juice agar plates, flies were allowed to lay eggs twice for 2 h in order to reduce egg retention. After 2-h egg collection intervals at 25°C, eggs were transferred to petri dishes containing fresh yeast paste and reared at 25°C. Pupariation was scored in 2-h intervals. For the hsRNAi experiments, larvae were reared on yeast until the late L2 stage, at which *w^1118^* controls and *hsDHR4-RNAi* L2 larvae were heat shocked for 35 min at 37.5°C. After a 4-h recovery, newly molted L3 larvae were transferred to yeast paste supplemented with 0.05% bromophenol blue to monitor their gut clearing status [71],[72].

### qPCR

All qPCR data shown here are based on 3–4 biological samples each tested in triplicate. Whole larvae were collected in distilled water and snap-frozen in liquid nitrogen, while dissected tissue samples were prepared in ice-cold PBS, rinsed twice with fresh PBS, transferred to TRIzol (Invitrogen), and snap-frozen in liquid nitrogen. Total RNA of whole larvae was isolated following a modified TRIzol protocol, where we substituted sodium acetate with lithium chloride for RNA precipitation. Total RNA from tissue samples was extracted using the RNAqueous-Micro Kit (Ambion) following the manufacturer's instruction. RNA samples (0.5–2 µg/reaction) were reverse transcribed using ABI High Capacity cDNA Synthesis kit, and the synthesized cDNA was used for qPCR (StepOnePlus, Applied Biosystems) using PowerSYBR Green PCR master mix (Applied Biosystems) with 5 ng of cDNA template with a primer concentration of 200 nM. Samples were normalized to *rp49* based on the ΔΔCT method. All primer sequences can be found in Table S1.

### Transgenic Constructs

To generate *pUAST-DHR4* cDNA, a 6.3 kb fragment containing the full-length synthetic cDNA of *DHR4* [35] was cut with *Eco* RI and *Xba* I from Litmus 28 and cloned into *pUAST* digested with the same enzymes. For the *pUAST-DHR4*-RNAi construct, the same inverted repeat used in the *hsDHR4*-RNAi [35] was used to clone the fragment into *pUAST* using *Xba* I for all restriction cuts. Transgenic flies were generated by injecting DNA at a concentration of 0.5 µg/µl along with 0.1 µg/µl helper plasmid pΔ2–3 into embryos following standard procedures [73],[74].

### Immunostaining

Tissues were dissected from larvae in PBS, fixed in 4% paraformaldehyde (EMS #15710) in PBST (PBS containing 0.3% Triton-X 100) for 20 min at room temperature (RT), and washed in PBST. Tissues were then blocked for 2 h at RT or overnight at 4°C in PBST/5% NGS. Primary antibodies were incubated at 4°C overnight, while the secondary antibody was either incubated overnight at 4°C or 4 h at RT. Nuclei were stained with DAPI (1∶5000). After several wash steps, tissues were mounted in SlowFade Gold Antifade Reagent (Invitrogen). Images were captured on a Nikon C1 plus confocal microscope. Anti-DHR4 antibody was used at a dilution of 1∶500, and anti-ERK antibody was used at a dilution of 1∶100 (Cell Signaling #4695). Secondary antibodies (anti-rabbit Cy3) were used at a dilution of 1∶200 (Rockland #611-104-122).

### Ring Gland Microarrays

*DHR4*-RNAi and *w^1118^* populations were heat shocked as late L2 larvae for 35 min at 37.5°C. To carefully stage larvae at the L2/L3 molt, L3 larvae were discarded 4 h after the heat treatment, and L3 larvae that molted in the following hour were allowed to feed for either 4 h or 8 h before their ring glands were dissected in ice-cold PBS. Ten ring glands were dissected and washed twice in PBS before being transferred to ice-cold TRIzol reagent (Invitrogen). The lysates were then vortexed for 5 s at RT, flash frozen, and stored at −80°C. Total RNA was isolated by Ambion RNAqueous-Micro Kit. Isolated RNA was quantified by RiboGreen Quanti Kit (Invitrogen) and RNA integrity was analyzed by Agilent Bioanalyzer Pico Chips. RNA linear amplification was based on the MessageII RNA Amplification kit (Ambion): First-strand cDNA synthesis was carried out by a T7-(dT) primer and ArrayScript reverse transcriptase using 50 ng RNA of each ring gland sample. Second-strand cDNA synthesis was performed according to the provided protocol. Purified cDNA was then fed into the IVT reactions. The amplified RNA (aRNA) was column-purified and analyzed by Agilent Bioanalyzer Nano chips. 1 µg of aRNA was used for double-stranded cDNA synthesis (Invitrogen SuperScript One-Cycle cDNA Kit) and 1 µg of the purified cDNA was Cy3-labeled by Roche NimbleGen one-color cDNA labeling kit. From this, 4 µg of Cy3-labeled cDNA was hybridized on a *Drosophila* 12×135K Array (NimbleGen). Each condition was analyzed by three independent biological samples. Chip hybridization and scanning was performed by the Alberta Transplant Applied Genomics Center. Raw data were normalized with the NimbleScan software (NimbleGen) using the RMA algorithm [75], and data were analyzed with Arraystar 4.0 (DNAstar) as well as Access (Microsoft).

### Ecdysteroids Measurements

Larvae were collected in 1.5 ml tubes and stored at −80°C. Samples were then homogenized in methanol and centrifuged at maximum speed, after which the precipitates were re-extracted with ethanol. The extracts were pooled and dried with a SpeedVac centrifuge. The dried extracts were thoroughly dissolved in EIA buffer at 4°C overnight prior to the EIA assay. 20E EIA antiserum (#482202), 20E AChE tracer (#482200), Precoated (Mouse Anti-Rabbit IgG) EIA 96-Well Plates (#400007), and Ellman's Reagent (#400050) were all purchased from *Cayman* Chemical, and assays were performed according to the manufacturer's instructions.

#### Sterol rescue experiments

We used two types of media, standard medium and instant food. For 20E-containing standard medium, 33 mg of 20E (Steraloids Inc.) was dissolved in 100 ml of 6% ethanol, of which ∼2 ml was added to 1 g of dry baker's yeast for a single plate. Control food contained 6% ethanol w/o 20E. 30∼40 larvae were raised on each yeast plate in order to avoid overpopulation. For the rescue experiment of *Cyp6t3*-RNAi larvae with ecdysteroid precursors, we used an instant fly medium (“4–24,” Carolina Biological Supply Company, hereafter referred to as C424), which is naturally low in cholesterol and other sterols [50]. Ecdysteroid precursors were dissolved in 100% ethanol, and added to each vial with 1 g C424 powder. Ethanol was allowed to evaporate completely before the medium was mixed vigorously with 5 ml of distilled water. The final concentrations for the precursors used were: cholesterol: 20 µg/ml and 7-dehydrocholesterol: 100 µg/ml, 5β-ketodiol: 200 µg/ml, E: 40 µg/ml, 20E: 200 µg/ml. 5β-ketodiol was a kind gift from Ryusuke Niwa; all other sterols were purchased from Steraloids Inc.

### *In situ* RNA Hybridization

DIG-labeled RNA probes were generated by in vitro transcription following the manufacturer's instructions (Roche DIG RNA Labeling Mix, #11 277 073 910). L3 larvae were dissected in ice-cold PBS and fixed in 4% paraformaldehyde for 20 min at RT. After treatment with 1% H_2_O_2_, samples were stored in hybridization buffer at −20°C. Tissues were pre-hybridized in hybridization buffer for 3 h at 58°C and RNA probes were denatured for 3 min at 80°C h. Probe hybridization was performed for 18 h at 58°C, followed by extensive wash steps at 58°C. After cooling, tissues were blocked with PBTB buffer (2% NGS and 1% BSA) for 1 h at RT before overnight incubation with mouse Anti-Digoxin antibody (Jackson ImmunoResearch Cat. # 200-062-156, 1∶500 dilution) at 4°C. Tissues were then incubated with streptavidin-HRP conjugates (Molecular Probes #S991, 1∶400 dilution) in PBTB for 1 h at RT, followed by six wash steps (1 h each) in PBTB at RT. Before TSA amplification, tissues were washed in PBTB. Tyramide reagents (PerkinElmer TSA Plus Cyanine 3 Kit, Cat. #NEL744001KT) were diluted 1∶1000 in 1× amplification buffer provided by the kit. TSA reactions were performed for 40 min at RT and washed 6 times for 1 h in PBS at RT. Tissues were mounted in the ProLong Gold antifade reagent (Invitrogen, P36934) and analyzed by confocal microscopy (Nikon AZ-C1 Confocal Microscope System).

## Supporting Information

Figure S1 Schematic representation of *DHR4* expression profiles during *Drosophila* larval development. *DHR4* is expressed in the prothoracic gland (red) throughout larval development, but fat body expression of *DHR4* (brown) only occurs prior to molts and during puparium formation. *y*-axis represents relative 20E titers. This idealized ecdysone curve represents data from several papers [5],[76],[77]. The tissue-specific expression of DHR4 represents data from this article and our previous report about DHR4 [35].
(PDF)

Figure S2 qPCR analysis of *Sgs-4* transcripts levels in larvae heat treated in early *hsDHR4*-RNAi L3 larvae. *Sgs-4* transcripts levels of *hsDHR4*-RNAi (grey) animals with RNAi treatment in early L3 were analyzed by qPCR. Hours are relative to the L2/L3 molt. All fold changes were normalized to 16-h time point in controls (black). Error bars represent 95% confidence intervals.
(PDF)

Figure S3 Time course qPCR analysis of *E74* transcripts levels in larvae heat treated in late L2 or early L3. *E74A* (upper panels) and *E74B* (bottom panels) transcripts levels were plotted as hours relative to the L2/L3 molt, and all fold changes were calibrated to either the control 0-h time point (left panels) or the 8-h time point (right panels). Circles represent controls (*w^1118^*) and squares stand for *hsDHR4*-RNAi larvae. Left panels: L2 larvae received heat shock ∼4 h before the molt to L3. Right panels: L3 larvae were heat shocked 4 h after the L2/L3 molt. Error bars represent 95% confidence intervals.
(PDF)

Figure S4 *Cyp6t3* transcripts are specifically enriched in the ring gland. qPCR analysis of *Cyp6t3* mRNA levels in ring gland (RG, grey bar) versus whole body (WB, black bar) isolated at 4 h after the molt. RNA from *w^1118^* ring glands and total larvae were linearly amplified before qPCR analysis. Error bars represent 95% confidence intervals.
(PDF)

Figure S5 Time course qPCR analysis in early *w^1118^* L3 larvae. Brain-ring gland complexes were isolated from carefully staged animals, and qPCR was carried out to measure relative mRNA levels of selected cytochrome P450 genes in *w^1118^* at 0, 4, 8, and 12 h after the L2/L3 molt. All fold changes were normalized to the 0-h time point. Error bars represent 95% confidence intervals.
(PDF)

Figure S6 Phenotypic characterization of *Cyp6t3*-RNAi line #30896 (VDRC). (A) Time course of puparium formation in *phm22>Cyp6t3*-RNAi (orange) and *phm22* x *w^1118^* control lines (black). Error bars reflect standard deviation, which are based on three replicates. (B) 20E rescues large pupal phenotype. The developmental delay shown in (A) results in larger *phm22>Cyp6t3*-RNAi animals (3^rd^ pupa from left) while *phm22* x *w^1118^* controls are normal sized (left pupa). Supplementing media with 20E results in normal-sized controls and *phm22>Cyp6t3*-RNAi animals. *N* = 180 for each condition.
(PDF)

Figure S7 Whole-body ecdysteroid titers for *Cyp6t3* RNAi embryos and larvae. (A) Whole-body ecdysteroid titers for controls (*phm22>w^1118^*, grey) and *phm22>Cyp6t3*-RNAi animals (striped). Left: embryos (in pg/100 embryos). Middle: L1 larvae (in pg/100 larvae). Right: L2 larvae (in pg/larva) at different time points as indicated. Error bars indicate standard error. * *p*<0.01. In L2, the ecdysone pulse occurs in the 6–12 h time window, while the 18–24 h window lies outside the peak.
(PDF)

Figure S8 Sequence alignment of *Drosophila melanogaster* Cyp6t3 with related proteins from a variety of species. Cyp6t3 from *Drosophila melanogaster* (*Dm*) was aligned with the most highly conserved proteins from representatives of Diptera (*Drosophila pseudoobscura*, *Dpse*; *Aedes aegypti*; *Aa* and *Anopheles gambiae*; *Ag*), Lepidoptera (*Bombyx mori*; *Bm*), Coleoptera (*Tribolium castaneum*; *Tc*), and Hymenoptera (*Apis mellifera*; *Am*), as well as the *Dm* protein Phantom (a less closely related member of the Cyp306 family). The sequences of *Dm* Cyp6t3, *Dpse* Cyp6t3, and *Dm* Phm were determined using Flybase. *Dm* Cyp6t3 was used as the query for a BLASTp search specific for each of the organisms indicated above, and all the top hits along with Dm Phm and Dpse Cyp6t3 were aligned using ClustalW2 (Gonnet weight Matrix, gap penalty N = 10, gap extension penalty N = 0.2). BoxShade was used to shade the resultant alignment. Dark shading indicates a residue that shares identity with >60% of the aligned residues at that position, while the light shading indicates >60% similarity.
(PDF)

Figure S9 Phylogenetic tree of *Drosophila melanogaster* Cyp6t3 with related proteins from a variety of species. The aligned proteins were assembled into a phylogenetic tree using SeaView [65]. To construct the tree, the parsimony algorithm was used and bootstrapped with 1,000 replicates. Numbers indicate the support values for each node.
(PDF)

Table S1 A list of all primers used for qPCR and *in situ* hybridization.
(PDF)

## Abbreviations

20E: 20-Hydroxyecdysone
7dC: 7-dehydrocholesterol
*DHR4*: *Drosophila Hormone Receptor 4*
*dib*: *disembodied*
E: ecdysone
L1: first instar
L2: second instar
L3: third instar
PG: prothoracic gland
*phm*: *phantom*
PTTH: prothoracicotopic hormone
qPCR: quantitative real-time PCR
RG: ring gland
RNAi: RNA interference
*sad*: *shadow*
*shd*: *shade*
