# Supplementary figures and images for: Nuclear Receptor DHR4 Controls the Timing of Steroid Hormone Pulses During Drosophila Development
Source: PLoS Biol. 2011 Sep 27;9(9):e1001160. doi: 10.1371/journal.pbio.1001160 (PMC3181225; doi:10.1371/journal.pbio.1001160)

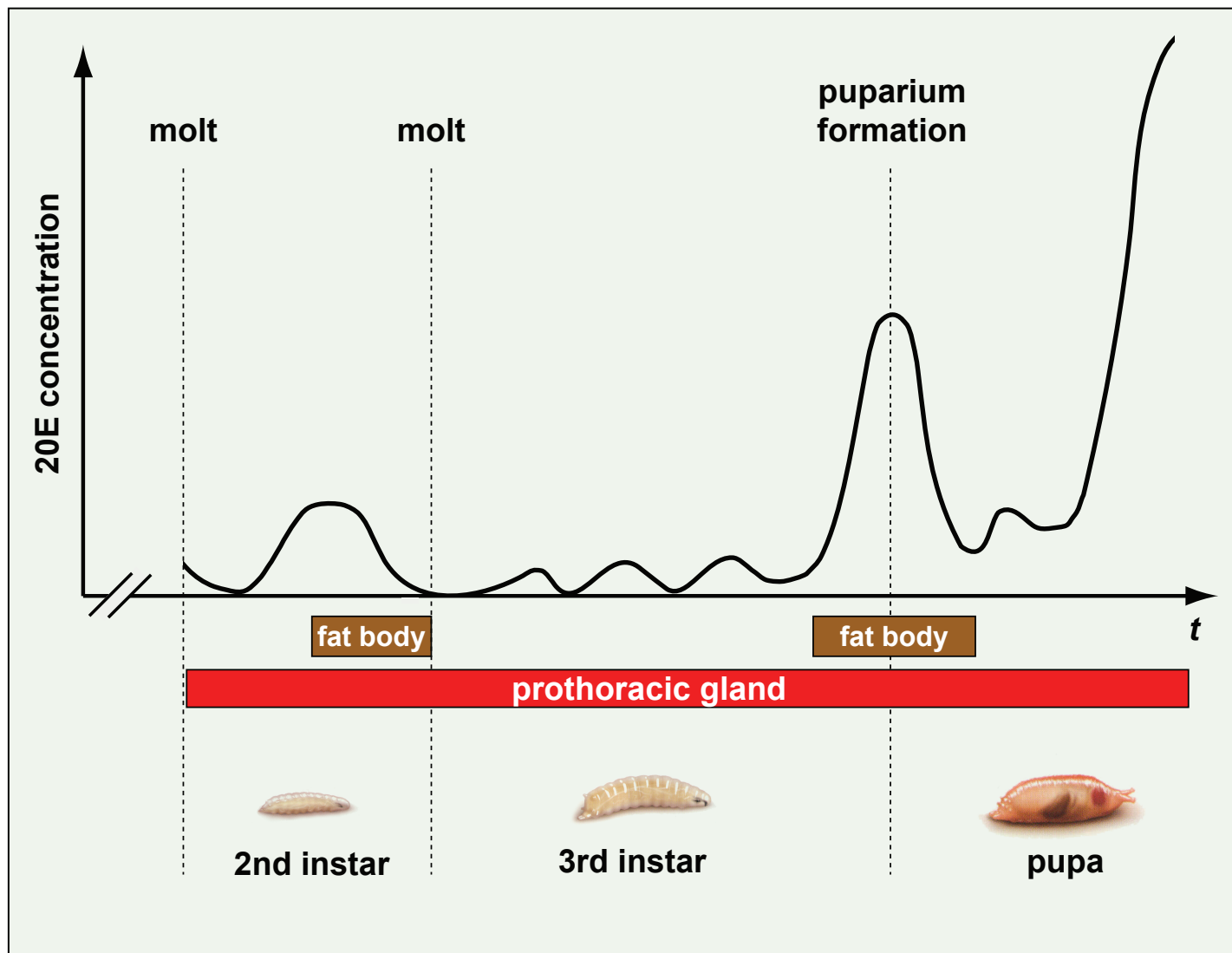

Supplement: Figure S1 — Schematic representation of DHR4 expression profiles during Drosophila larval development. DHR4 is expressed in the prothoracic gland (red) throughout larval development, but fat body expression of DHR4 (brown) only occurs prior to molts and during puparium formation. y-axis represents relative 20E titers. This idealized ecdysone curve represents data from several papers [5],[76],[77]. The tissue-specific expression of DHR4 represents data from this article and our previous report about DHR4 [35]. (PDF) [file pbio.1001160.s001.pdf]

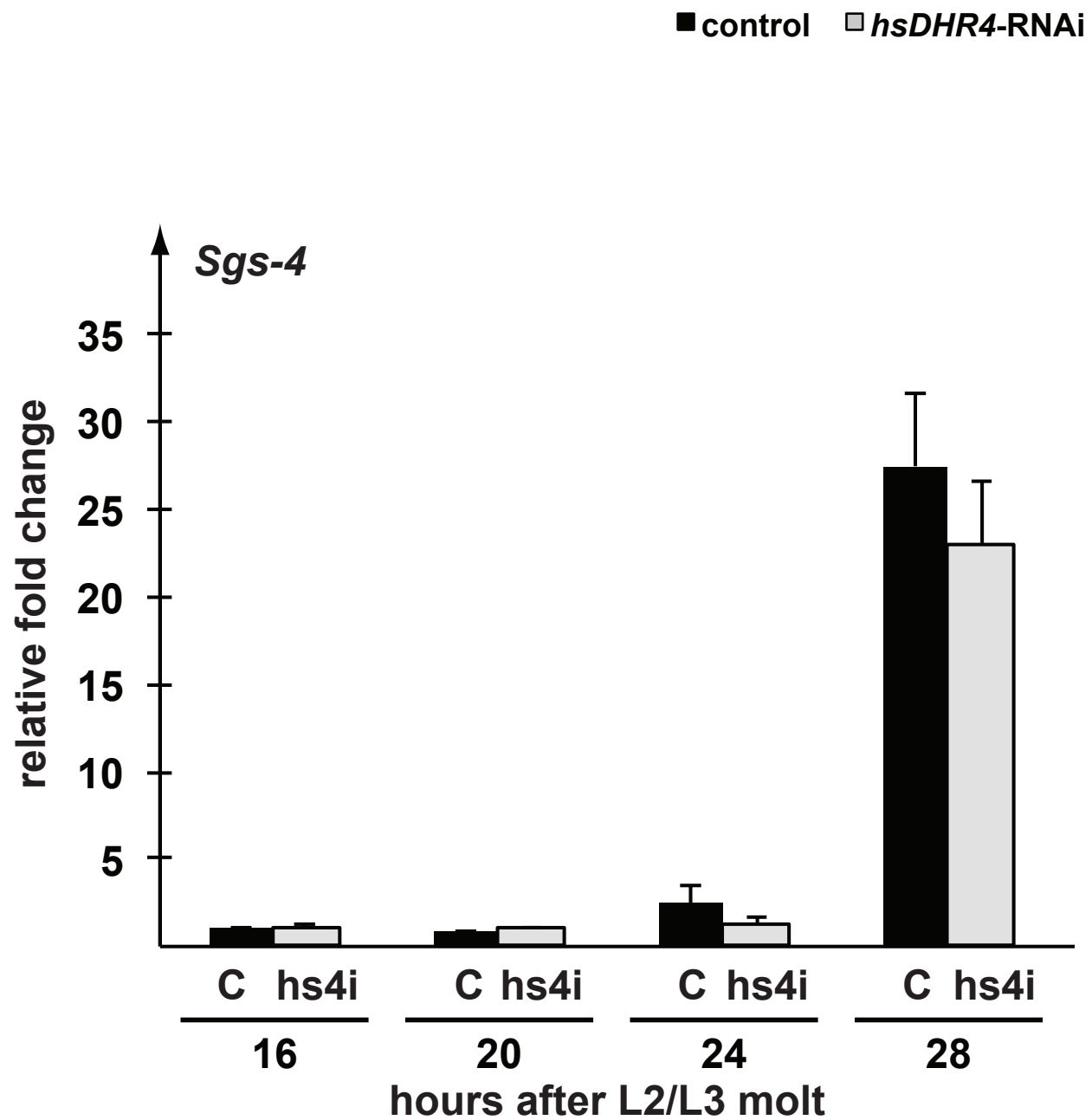

Supplement: Figure S2 — qPCR analysis of Sgs-4 transcripts levels in larvae heat treated in early hsDHR4-RNAi L3 larvae. Sgs-4 transcripts levels of hsDHR4-RNAi (grey) animals with RNAi treatment in early L3 were analyzed by qPCR. Hours are relative to the L2/L3 molt. All fold changes were normalized to 16-h time point in controls (black). Error bars represent 95% confidence intervals. (PDF) [file pbio.1001160.s002.pdf]

## HS: Late second instar

## HS: Early third instar

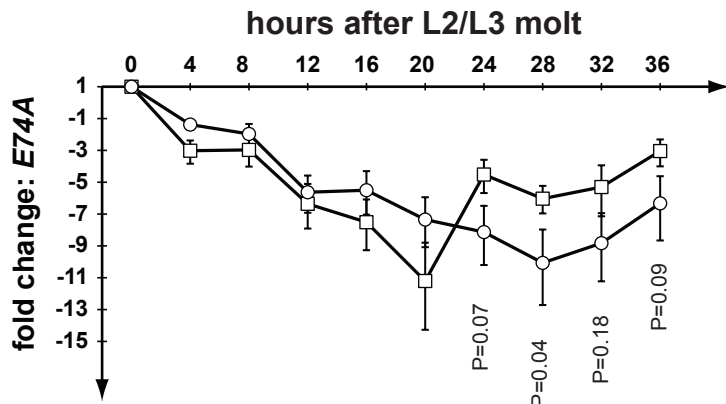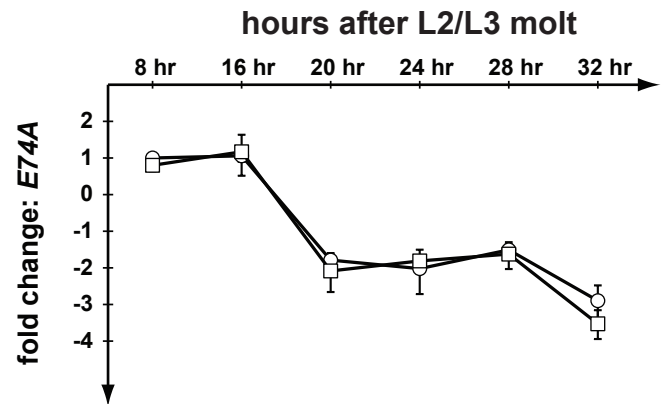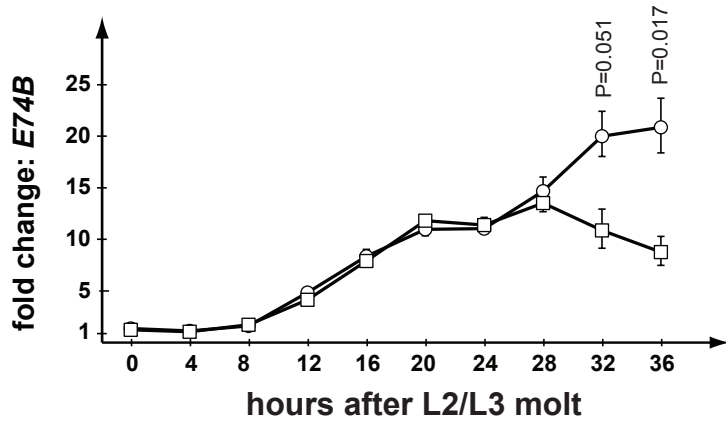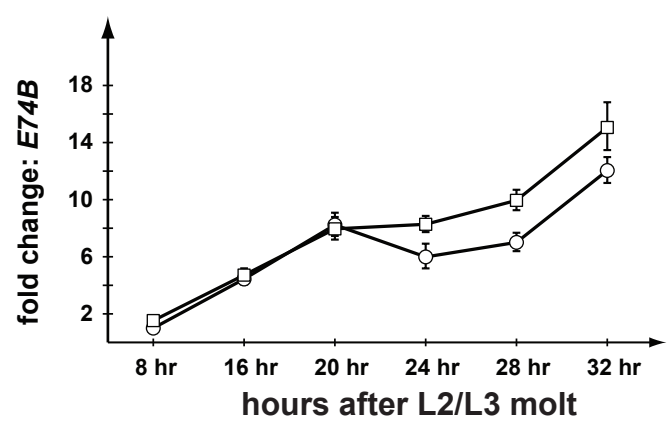

—○— *w1118*

—□— *hsDHR4-RNAi*

Supplement: Figure S3 — Time course qPCR analysis of E74 transcripts levels in larvae heat treated in late L2 or early L3. E74A (upper panels) and E74B (bottom panels) transcripts levels were plotted as hours relative to the L2/L3 molt, and all fold changes were calibrated to either the control 0-h time point (left panels) or the 8-h time point (right panels). Circles represent controls (w1118) and squares stand for hsDHR4-RNAi larvae. Left panels: L2 larvae received heat shock ∼4 h before the molt to L3. Right panels: L3 larvae were heat shocked 4 h after the L2/L3 molt. Error bars represent 95% confidence intervals. (PDF) [file pbio.1001160.s003.pdf]

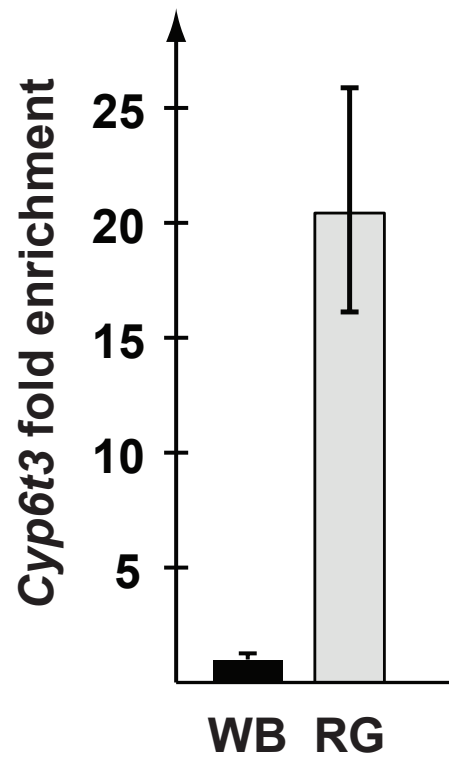

Supplement: Figure S4 — Cyp6t3 transcripts are specifically enriched in the ring gland. qPCR analysis of Cyp6t3 mRNA levels in ring gland (RG, grey bar) versus whole body (WB, black bar) isolated at 4 h after the molt. RNA from w1118 ring glands and total larvae were linearly amplified before qPCR analysis. Error bars represent 95% confidence intervals. (PDF) [file pbio.1001160.s004.pdf]

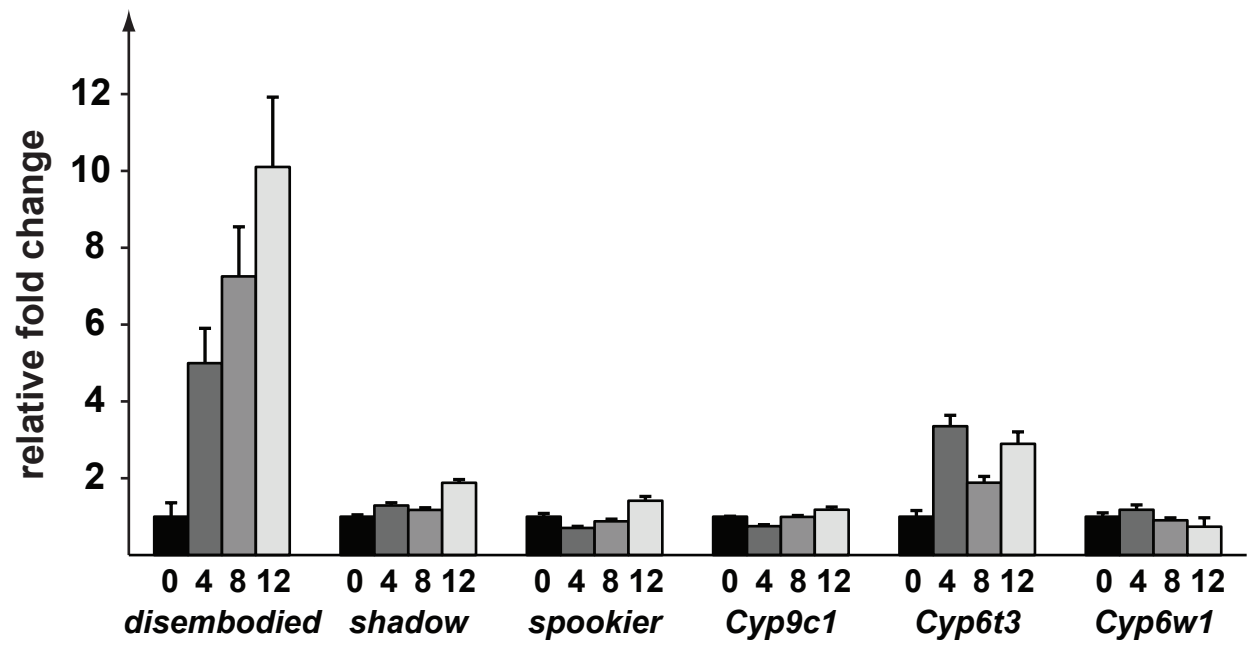

Supplement: Figure S5 — Time course qPCR analysis in early w1118 L3 larvae. Brain-ring gland complexes were isolated from carefully staged animals, and qPCR was carried out to measure relative mRNA levels of selected cytochrome P450 genes in w1118 at 0, 4, 8, and 12 h after the L2/L3 molt. All fold changes were normalized to the 0-h time point. Error bars represent 95% confidence intervals. (PDF) [file pbio.1001160.s005.pdf]

**A**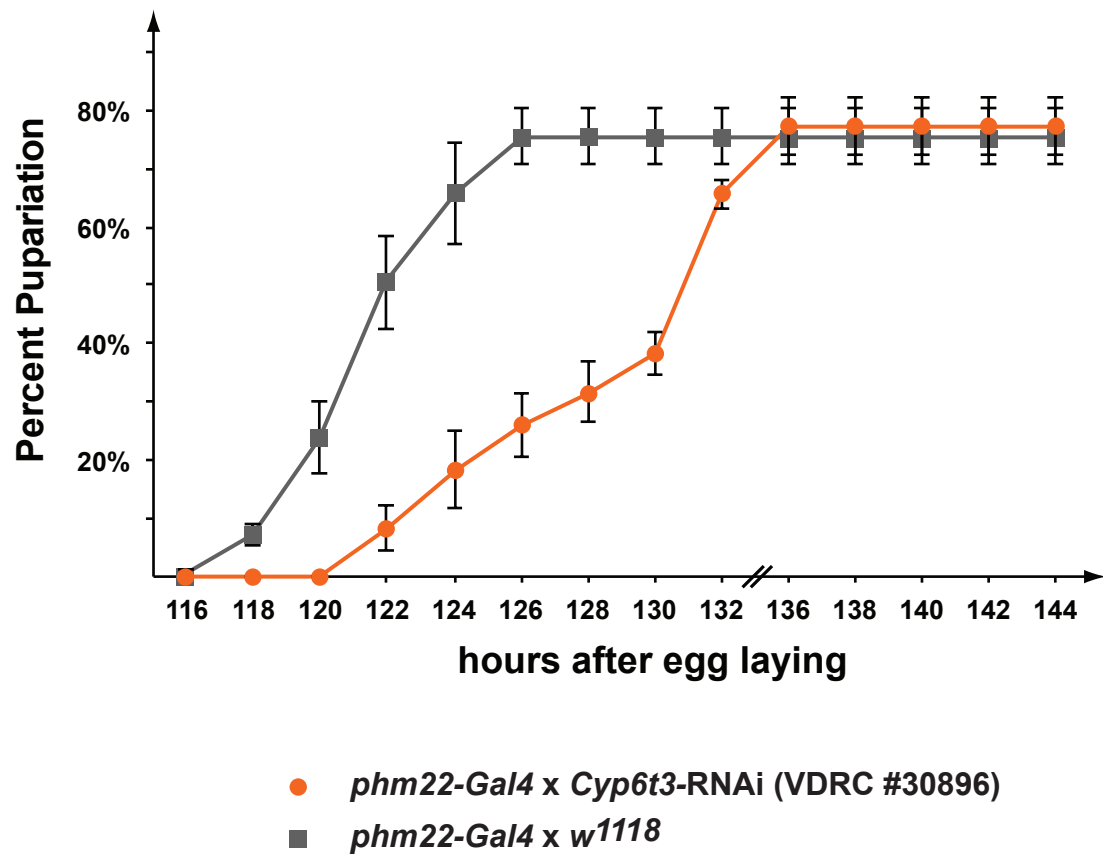**B**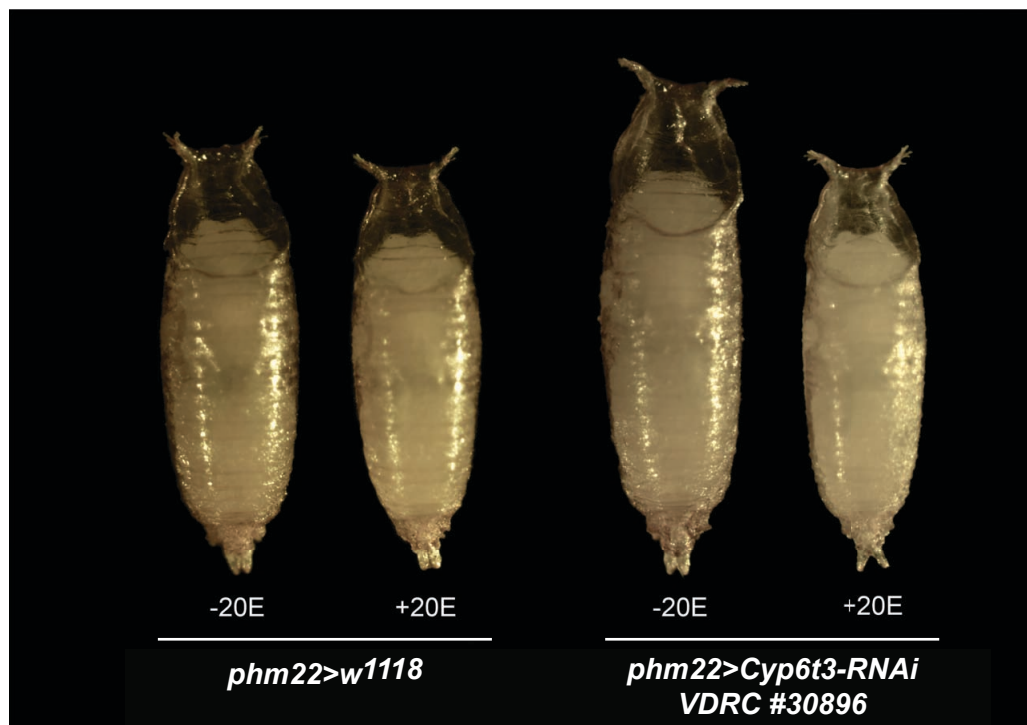

Supplement: Figure S6 — Phenotypic characterization of Cyp6t3-RNAi line #30896 (VDRC). (A) Time course of puparium formation in phm22>Cyp6t3-RNAi (orange) and phm22 x w1118 control lines (black). Error bars reflect standard deviation, which are based on three replicates. (B) 20E rescues large pupal phenotype. The developmental delay shown in (A) results in larger phm22>Cyp6t3-RNAi animals (3rd pupa from left) while phm22 x w1118 controls are normal sized (left pupa). Supplementing media with 20E results in normal-sized controls and phm22>Cyp6t3-RNAi animals. N = 180 for each condition. (PDF) [file pbio.1001160.s006.pdf]

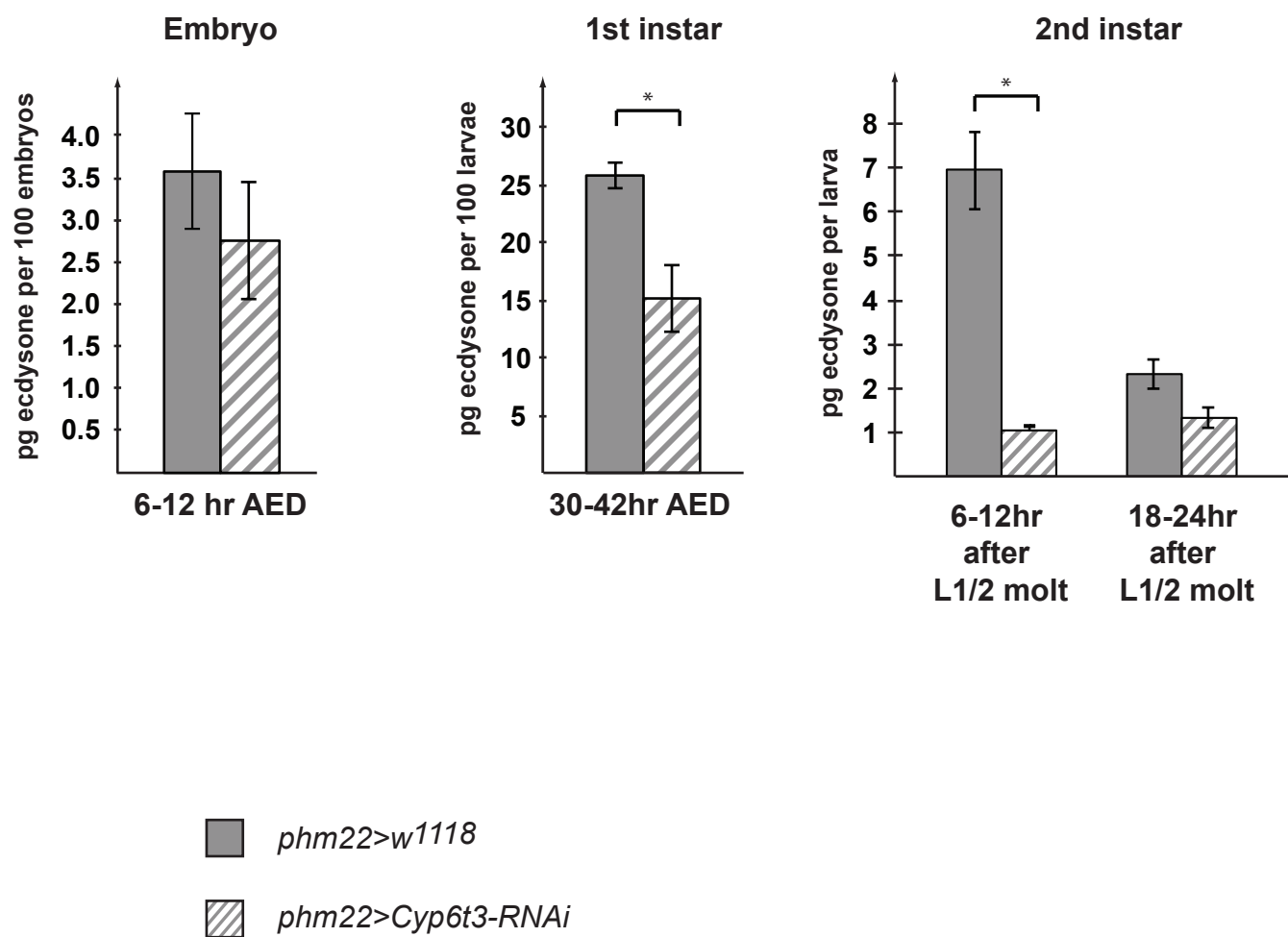

Supplement: Figure S7 — Whole-body ecdysteroid titers for Cyp6t3 RNAi embryos and larvae. (A) Whole-body ecdysteroid titers for controls (phm22>w1118, grey) and phm22>Cyp6t3-RNAi animals (striped). Left: embryos (in pg/100 embryos). Middle: L1 larvae (in pg/100 larvae). Right: L2 larvae (in pg/larva) at different time points as indicated. Error bars indicate standard error. * p<0.01. In L2, the ecdysone pulse occurs in the 6–12 h time window, while the 18–24 h window lies outside the peak. (PDF) [file pbio.1001160.s007.pdf]

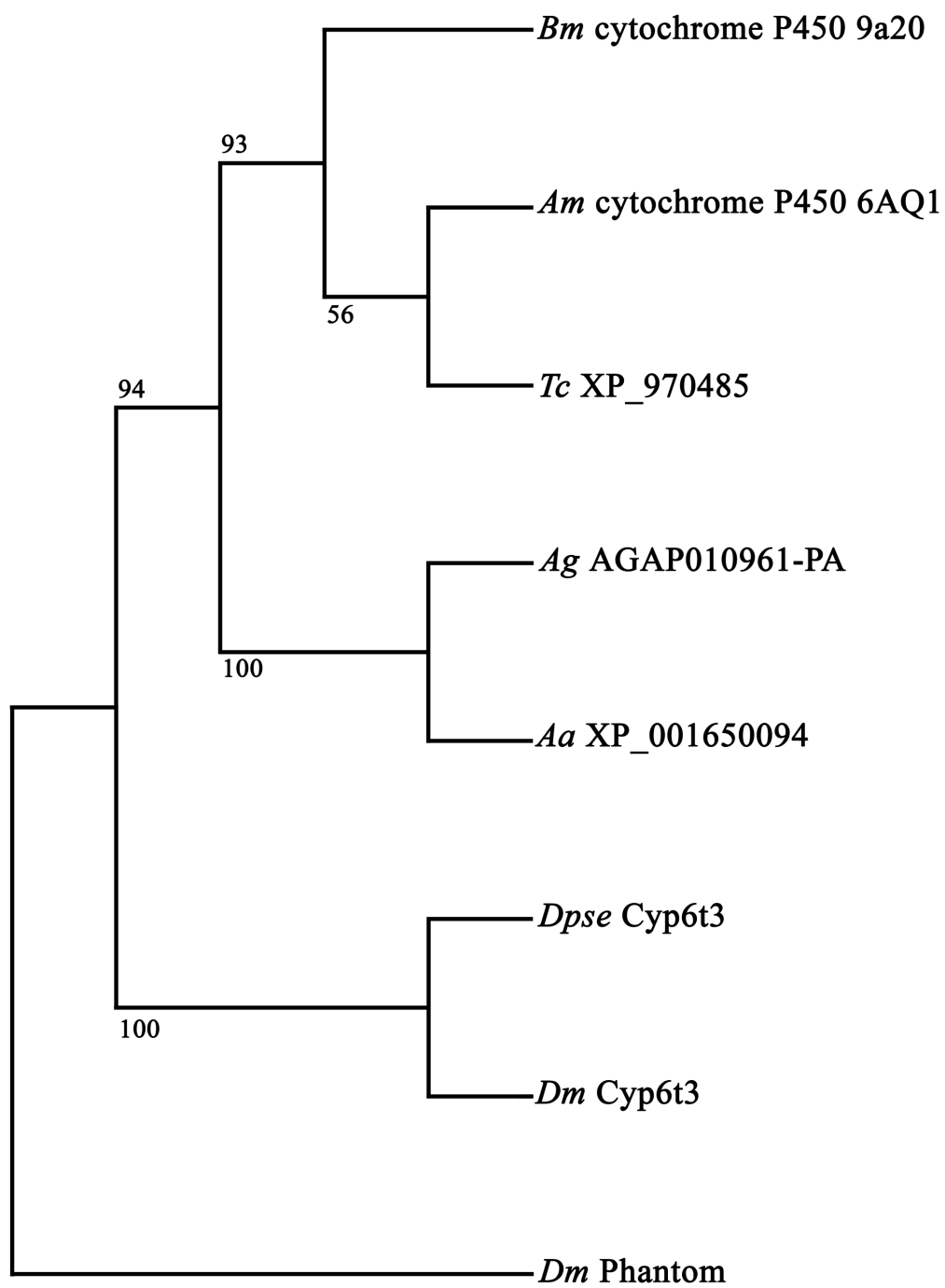

Supplement: Figure S9 — Phylogenetic tree of Drosophila melanogaster Cyp6t3 with related proteins from a variety of species. The aligned proteins were assembled into a phylogenetic tree using SeaView [65]. To construct the tree, the parsimony algorithm was used and bootstrapped with 1,000 replicates. Numbers indicate the support values for each node. (PDF) [file pbio.1001160.s009.pdf]
