# Supplementary material for: Nuclear Receptor DHR4 Controls the Timing of Steroid Hormone Pulses During Drosophila Development
Source: PLoS Biol. 2011 Sep 27;9(9):e1001160. doi: 10.1371/journal.pbio.1001160 (PMC3181225; doi:10.1371/journal.pbio.1001160)
Supplement: Figure S8 — Sequence alignment of Drosophila melanogaster Cyp6t3 with related proteins from a variety of species. Cyp6t3 from Drosophila melanogaster (Dm) was aligned with the most highly conserved proteins from representatives of Diptera (Drosophila pseudoobscura, Dpse; Aedes aegypti; Aa and Anopheles gambiae; Ag), Lepidoptera (Bombyx mori; Bm), Coleoptera (Tribolium castaneum; Tc), and Hymenoptera (Apis mellifera; Am), as well as the Dm protein Phantom (a less closely related member of the Cyp306 family). The sequences of Dm Cyp6t3, Dpse Cyp6t3, and Dm Phm were determined using Flybase. Dm Cyp6t3 was used as the query for a BLASTp search specific for each of the organisms indicated above, and all the top hits along with Dm Phm and Dpse Cyp6t3 were aligned using ClustalW2 (Gonnet weight Matrix, gap penalty N = 10, gap extension penalty N = 0.2). BoxShade was used to shade the resultant alignment. Dark shading indicates a residue that shares identity with >60% of the aligned residues at that position, while the light shading indicates >60% similarity. (PDF) [file pbio.1001160.s008.pdf]

```
Dm Cyp6t3                 1 -----------MVSLWLLMLGILSIYLWLRQRYSYFERHNIVHLPVSAWTPLGHLRKLLLLRISFGDLFHNIYADPRGGQ
Dpse Cyp6t3               1 -----------MLLIWLLLLTIVTLNFWLRHKYDYFRSRGIPHLPPSSWSPMGNLGQLLFLRISFGDLFRQLYADPRNGQ
Ag AGAP010961-PA           1 ---IMSLEVVDLLYTVALLLLYLGWRKQT----YWTRAGVPYIPEI--PIIGNFATIFLQRHSSFEYIEHIYHHARTRT
Am cytochrome P450 6AQ1   1 MIYVNSDALTGYLTLVLVCLAGFAVWFLSTSGTRYWQFFRVPYIEGR--PLVGNFLDAFLFRKSTFELMDALYQNPKVEG
Aa XP_001650094           1 --MNLLTPYWSLDILIVSSSLMIAVYLYASWKLKYWSRRGIMQITPS--PLFGNFKKCILFQKSVSEIIRELY--GQNEG
Tc XP_970485              1 ---MLLTPYLPLDTVVLLSVLALLLYKYFSRNFDHWEKKNVFYFKPI--PFFGNFVDISLFRTTIGEHLAKLY--NQTT-
Bm cytochrome P450 9a20   1 -----------MILLIWAVVLIAAFVLFYKQAYSLFSKHGVKGFTPL--PFFGNMGRIVIKMDHFSDHIQSLY--DSFPE
Dm Phantom                1 MSADIVDIGHTGWMPSVQSLSILLVPGALVLVILYLCERQCNDLMGAPPGPWGLPFLGYLPFLDARAPHKSLQKLAKRY

Dm Cyp6t3                70 AKVVGFFLFQTPALMVRDPELIRLVLIKHFNSFLNRYEAADAAGDPMGSLTLPLAKYHNWRESR----------------
Dpse Cyp6t3              70 AKIVGFFIFQTPALMVRDPELIRQVLIKNFNNFLNRFESADAG-DPMGALTLPLAKYHHWKESR----------------
Ag AGAP010961-PA          72 SDFFGVNIFTRPALVLRSPELIKRLTCTDAQRFINRQMCTDPFHDAIGYFNLIMIREPLWKQLR----------------
Am cytochrome P450 6AQ1  79 TKLFGVSIMMQPALVIRDPELVKQVLIKDATYFSNRYMCTDRIHDAIGYYNLLMIKNPHWKQLR----------------
Aa XP_001650094          75 LPFMGFYIFYKPFFLVRDIELVKHILVKDFNTFANKHTSADSKNDRIGYSNLFIIKNPAWKYLR----------------
Tc XP_970485             73 EPFFGIFVFDKPHLIIKSPELVKTILVRDFNNFDDRCIAS-PHHDPLVKNMLFLNKNPEWKNVR----------------
Bm cytochrome P450 9a20  66 ERFVGRYEFLNPMVIIRDIELLKKITVKDFEHFLDHRTIINKDTDPFFGRSLFFLRDQDWKDMR----------------
Dm Phantom               81 GGIFELKMGRVPTVVLSDAALVRDFFRR---DVMTGRAPLYLTHGIMGGFGICAQEDIWRHARRETIDWLKALGMTRRP

Dm Cyp6t3               134 ---------------QSMAQLFTSGRMRESMYPLMMG---------VVIDLEQYLQRKLGDRSE--RMLPLSKMCQLYTT
Dpse Cyp6t3             133 ---------------QCMSQLFTSGRMRDVMYSQMLD---------VASDLEQYLNRKLGDRLE--RVLPLGRMCQLYTT
Ag AGAP010961-PA         136 ---------------GFLSPSVTSAKLKRMQPLIEQKLIALVTTFQVGADMMASLDALPVLRPN-VRETEFKELCARFTT
Am cytochrome P450 6AQ1 143 ---------------AYLTPSLSLNKIKRMYSLVDQ----------IGNNMLAHLDGVQQKPTK-LRETEFKELCARFTT
Aa XP_001650094         139 ---------------GKLTSVFTSGKLKKMFDLMLI----------IGKNLEKHLELLNLDGNG--KEVELKDLCANFTT
Tc XP_970485            136 ---------------VKMTPVFTTGKLKGMIPLIND----------VGETMTKYIAQKIPN-----FSLEAKEICAKFST
Bm cytochrome P450 9a20 130 ---------------STLSPAFTSSKMKLMMPFIVE----------VGEQMNKALKQRIQEAGVGYVDIDSKDLTTRYAN
Dm Phantom              158 GELRARLERRIARGVDECVRLFDTEAKKSCASEVNPLPALHHSLGNIINDLVFGITYKRDDPDWLYLQRLQEEGVKLIGV

Dm Cyp6t3               188 DVTGNLFYSMDVGGLRRGKSQLLKKTKELFDPDPRKVLDFMSIFFLPQWTNLLRAKVFSEEYAQFMRGLVGHPRER----
Dpse Cyp6t3             187 DVTGNLFYSLNVGGLRRGRSELITKTKELFNTNPRKVLDFMSVFFLPKWTGVLKPKVFTEDYARYMRHLVDDHHEP----
Ag AGAP010961-PA         200 DVIASTFFGMQANCLKDEQSEFRYYGRKIFDYGPGRGLTMASFFFLPELVPYLRLKLFPRDTEAFLKAIIEQEIARREQT
Am cytochrome P450 6AQ1 197 DVIASTFFGIQANCLSDEESEFRYYGRKIFEYGPKRALNMAAFFFMPELVPYLGFKLFPRDTERFLKTIIEQEIARRETS
Aa XP_001650094         192 DLIGTTAFGVNLNSLKDPNSDFRENGRLVFDYNLKRAFEFFSIFFFPNLNKYFSVKFFGK-ATDYFRNSFWSVINQRIES
Tc XP_970485            186 DVIAKCAFGINANSFKNEDAEFRKIGRRIFDFRWSTAIQQTSYFFLPGLVNLLKFRMLDKDASDFLRETFWHTIKLREEK
Bm cytochrome P450 9a20 185 DVIASCAFGLKVDSITEENNQFYAMGKAASTFNFRQLLIFFGLASVPKLVKILRITLFQKEIKTFFRELILGTMKNREAQ
Dm Phantom              238 SGVVNFLPWLRHLPANVRNIRFLLEGKAKTHAIYDRIVEACGQRLKEKQKVFKELQEQKRLQRQLEKEQLRQSKEADPSQ

Dm Cyp6t3               264 --SKGDLIDQLLHLQSSR---------------PSSHYAQHP----------DFIASQASIILLAGFETSSALLGFTLYE
Dpse Cyp6t3             263 --TKGDLINQLQHFQLSR---------------SSNHYSQHP----------DFVASQAGIILLAGFETSSALMGFTLYE
Ag AGAP010961-PA         280 GESRGDFIDSMIALKNNN--------------ATLGVNEKILWISSSAALKGDILVAQAATFYMASFETTSSVLSFALYE
Am cytochrome P450 6AQ1 277 GENRGDFIDSMIALKNNE--------------ATIGVEEKIP-------LKGDILVAQAATFYMASFETTSSVLSFTLYE
Aa XP_001650094         271 NVKRNDLIDCLIELREK----------------HKNDESFEGFR-----FDGDDLVSQAAIFFTGGFETSSTTISFTLYE
Tc XP_970485            266 NLKANDLIDAIIALKD-------------------NQEFCKNMN-----FEGDKVVAQAAQFFVAGFETTSSTMAFTLYE
Bm cytochrome P450 9a20 265 NIIRPDMIHLLMEAKKGKLRHDEKSTKDSDAGFATVEESSVGKKDINRVWTDDDLVAQAVLFFVAGFETVSSAMTFLLHE
Dm Phantom              318 EQSEADEDDEESDEEDTYEP------------ECILEHFLAVRDTDSQLYCDDQLRHLLADLFGAGVDTSLATLRWFLLY

Dm Cyp6t3               317 LAKQPDLQKRLREELAEAFSLAPT-LTYETLV-TLPFLRKVCLEALRLYPAAAF-INRECTSS-DPNGFSLQPHVDFVVP
Dpse Cyp6t3             316 LAKAPDIQERLRSELREAFISTAT-LSYDTLM-TLPYLKMVCLEALRLYPAAAF-VNRECTSS-ASEGFSLQPHVDFIVP
Ag AGAP010961-PA         346 LTKNPHVQERLREEIRDIIVKYGREIPYEAYANEMPYLGMVISETNRLYPVLAF-IERQCTLPEGSTGYKLEPLHDYVIP
Am cytochrome P450 6AQ1 336 LTKNPEIQQRLREEIHNCIKKYGRDLSYECLVNEMPYLGMVISEAARLYPVLPF-IERQCSLPAGATGYKLDPFHNFVVP
Aa XP_001650094         330 LALNKDIQKTVRTEIHEALAQTDGKITYDMIT-NLPYLDMVVSETLRKYPPLGF-LDRVALHDYKIPNS------DVTID
Tc XP_970485            322 LCLQPQFQRRVRAEIATCLKEHNG-LTYEALQ-SMKYLNMCVCETLRKYPVLPF-LDRTCKEDYKLPNS------NVVIE
Bm cytochrome P450 9a20 345 LALNPEVQEKLVEEIRENEKNNNGKFDYNSIQ-NMVYLDMVVSEVLRLWPPVIA-LDRMCVKDYNLGKPNDKSKEDFIIR
Dm Phantom              386 LAR----EQRCQRRLHELLLPLGPSPTLEELE-PLAYLRACISETMRIRSVVPLGIPHGCKENFVVGDY--------FIK

Dm Cyp6t3               393 NGMPAYISILGLHRDEKYWPEPNRFDPERFDPDRSKDIIPMTYIPFGAGPHGCIGSRLGSLQLKLGLAHILRLCRVEVCE
Dpse Cyp6t3             392 PGMPAYISILGLHRDERFWPEPCVFDPERFGPERSRHIHPMTYIPFGAGPHGCIGSRLGVLQLKLGIVHILKQYWVETCE
Ag AGAP010961-PA         425 NGMPIMIPIYAIHRDPKYFPNPTVFDPERFAKENLDQIQPCTYMPFGVGPRTCLGSHFGTLQIKIALLKLLSQYRIERSP
Am cytochrome P450 6AQ1 415 NKMPVLVPIYAIHRDPKYFPDPLRFDPDRFSKDNADNIVPCSYMPFGVGPRTCLGSHFGTLQVKVAITRLLSKYRILRSE
Aa XP_001650094         402 KDTPVIIPMIAFHYDPKYFPNPEKYDPLRFSEEVKKTRPSYVYMPFGEGPHICIGMRLGLLQSKLGIIEILKDYEVSPCE
Tc XP_970485            393 KGTPVFIPMFGLHYDPQYFPNPQKYDPERFSDENMQNITPFSYIPFGEGPRNCIGERFGLIGTKLGLIHILSEFEVEKSS
Bm cytochrome P450 9a20 423 KDVAVGIPVWGLHRDPEFFPNPLKFDPERFSEENKHNIKPFSYMPFGLGPRNCIGSRFALCEVKVMTYQLLQHMEISPCE
Dm Phantom              453 GGSMIVCSEWAIHMDPVAFPEPEEFRPERFLTADGAYQAPPQFIPFSSGYRMCPGEEMARMILTLFTGRILRRFHLELPS

Dm Cyp6t3               473 --------QTVPQIRFNPKTFMLESKDEIYLRFYRDKL----
Dpse Cyp6t3             472 --------RTVSEIRFNPKSFMLESENEIYLRFCRSSL----
Ag AGAP010961-PA         505 --------STPEVLTYRKNAFTLNSNEGLYAALVEDAM----
Am cytochrome P450 6AQ1 495 --------SSPETLTYRKNAFTLHSNEGLYADLELDELC---
Aa XP_001650094         482 --------KTKIPMVLDPKGLTTTALGGLYLNIRKITIAAG-
Tc XP_970485            473 --------DTPVPLEFEPKSFVLASKVGLPMKFKKVMTSAA-
Bm cytochrome P450 9a20 503 --------KTCIPSKLSKETFNLRLEGGHWIRLKIRN-----
Dm Phantom              533 GTEVDMAGESGITLTPTPHMLRFTKLPAVEMRHAPDGAVVQD
```
