# Supplementary material for: Nuclear Receptor DHR4 Controls the Timing of Steroid Hormone Pulses During Drosophila Development
Source: PLoS Biol. 2011 Sep 27;9(9):e1001160. doi: 10.1371/journal.pbio.1001160 (PMC3181225; doi:10.1371/journal.pbio.1001160)
Supplement: Table S1 — A list of all primers used for qPCR and in situ hybridization. (PDF) [file pbio.1001160.s010.pdf]

Table S1. Primer pairs for qPCR and *in situ* probe

Gene	Primer Sequence
rp49	Forward 5' ttccttgacgtgccaaaact<br>Reverse 5'aatgatctataacaaaatcccctga
E74A	Forward 5' ccctttatcgacgatgcact<br>Reverse 5' acctccaacaagacgaccat
E74B	Forward 5' cgcgagttcaaagtgctcta<br>Reverse 5' ggagggagagtggtggtgt
Sgs4	Forward 5' aggcaagaagaacaccacca<br>Reverse 5' ttgctgtttagcaaccacctt
Cyp6t3	Forward 5' ggtgtgtttggaggcactg<br>Reverse 5' ggtgcactctctgttgacga
Cyp6t3 (in situ)	Forward 5' accatcactggaaggagagtcggc<br>Reverse 5' gaggaggtttcaaatccggccagc
Cyp6w1	Forward 5' aaaaacctcttctttgcacga<br>Reverse 5' tgtcctgcaagttctttcca
Cyp6a17-1	Forward 5' ggagcaggttgtgatggaa<br>Reverse 5' tcccttggcaatgaagtatttt
Cyp6a17-2	Forward 5' cacctacgagggaatcaagg<br>Reverse 5' tactttcgcagcgtttcca
Cyp9c1	Forward 5' tgggtaaagagtcgtacataaaaca<br>Reverse 5' tgaagactccatagaccttgtgc
phantom	Forward 5' ggcatcatgggtggattt<br>Reverse 5' caaggcctttagccaatcg
disembodied	Forward 5' gtgaccaaggagttcattagatttc<br>Reverse 5' ccaaaggtaagcaaacaggttaat
shadow	Forward 5' caagcggatatttgtagacttgg<br>Reverse 5' aacaaagcccactgactgct
spookier	Forward 5' cggtgatcgaaacaactcac<br>Reverse 5' cgagctaaatttctccgcttt
